# A multi-omic approach implicates novel protein dysregulation in post-traumatic stress disorder

**DOI:** 10.1186/s13073-025-01473-1

**Published:** 2025-04-29

**Authors:** Jiawei Wang, Yujing Liu, Hongyu Li, Tuan P. Nguyen, John Lee Soto-Vargas, Rashaun Wilson, Weiwei Wang, TuKiet T. Lam, Chi Zhang, Chen Lin, Traumatic Stress Brain Research Group, Victor E. Alvarez, David Benedek, Alicia Che, Dianne A. Cruz, David A. Davis, Ellen Hoffman, Bertrand R. Huber, Alfred Kaye, Adam T. Labadorf, Terence M. Keane, Mark W. Logue, Ann McKee, Brian Marx, Mark W. Miller, Crystal Noller, Janitza Montalvo-Ortiz, Meghan Pierce, William K. Scott, Paula Schnurr, Krista DiSano, Thor Stein, Robert Ursano, Douglas E. Williamson, Erika J. Wolf, Paul E. Holtzheimer, John H. Krystal, Keith A. Young, Matthew J. Girgenti, David A. Lewis, Jill Glausier, Paul E. Holtzheimer, Matthew J. Friedman, Kenneth R. Williams, Marina R. Picciotto, Angus C. Nairn, John H. Krystal, Ronald S. Duman, Keith A. Young, Hongyu Zhao, Matthew J. Girgenti

**Affiliations:** 1 Program of Computational Biology & Bioinformatics, Yale University, New Haven, CT 06511 USA; 2 Department of Psychiatry, Yale School of Medicine, New Haven, CT 06511 USA; 3 Department of Biostatistics, Yale School of Public Health, New Haven, CT 06510 USA; 4 NIDA Neuroproteomics Center, Yale School of Medicine, New Haven, CT 06511 USA; 5 Department of Molecular Biophysics and Biochemistry, Yale School of Medicine, New Haven, CT 06511 USA; 6 Keck MS & Proteomics Resource, Yale School of Medicine, New Haven, CT 06510 USA; 7 National Center for PTSD, United States Department of Veterans Affairs, White River Junction, VT 05009 USA; 8 Department of Psychiatry, University of Pittsburgh, Pittsburgh, PA 15213 USA; 9 Department of Psychiatry, Geisel School of Medicine at Dartmouth, Lebanon, NH 03756 USA; 10 Central Texas Veterans Health Care System, Research Service, Temple, TX 76504 USA; 11 Department of Psychiatry and Behavioral Sciences, Texas A&M University School of Medicine, Bryan, TX 77807 USA

**Keywords:** PTSD, Major depressive disorder, Multi-omics, MicroRNAs, Prefrontal cortex

## Abstract

**Background:**

Post-traumatic stress disorder (PTSD) is a common and disabling psychiatric disorder. PTSD involves multiple brain regions and is often comorbid with other psychiatric disorders, such as major depressive disorder (MDD). Recent genome-wide association studies (GWASs) have identified many PTSD risk loci and transcriptomics studies of postmortem brain have found differentially expressed genes associated with PTSD cases. In this study, we integrated genome-wide measures across modalities to identify convergent molecular effects in the PTSD brain.

**Methods:**

We performed tandem mass spectrometry (MS/MS) on a large cohort of donors (*N* = 66) in two prefrontal cortical areas, dorsolateral prefrontal cortex (DLPFC), and subgenual prefrontal cortex (sgPFC). We also coupled the proteomics data with transcriptomics and microRNA (miRNA) profiling from RNA-seq and small-RNA sequencing, respectively for the same cohort. Additionally, we utilized published GWAS results of multiple psychiatric disorders for integrative analysis.

**Results:**

We found differentially expressed proteins and co-expression protein modules disrupted by PTSD. Integrative analysis with transcriptomics and miRNA data from the same cohort pointed to *hsa-mir-589* as a regulatory miRNA responsible for dysregulation of neuronal protein networks for PTSD, including the gamma-aminobutyric acid (GABA) vesicular transporter, SLC32A1. In addition, we identified significant enrichment of risk genes for other psychiatric disorders, such as autism spectrum disorder (ASD) and major depressive disorder (MDD) within PTSD protein co-expression modules, suggesting shared molecular pathology.

**Conclusions:**

We integrated genome-wide measures of mRNA and miRNA expression and proteomics profiling from PTSD, MDD, and control (CON) brains to identify convergent and divergent molecular processes across genomic modalities. We substantially expand the number of differentially expressed genes and proteins in PTSD and identify downregulation of GABAergic processes in the PTSD proteome. This provides a novel framework for future studies integrating proteomic profiling with transcriptomics and non-coding RNAs in the human brain studies.

**Supplementary Information:**

The online version contains supplementary material available at 10.1186/s13073-025-01473-1.

## Background

Post-traumatic stress disorder (PTSD) is a severe mental illness that affects millions of people worldwide [1, 2]. Patients with PTSD typically have symptoms that include re-experiencing of traumatic memories [3, 4], hyperarousal, emotional numbing, dysphoric mood, and avoidance. In addition, PTSD is frequently comorbid with other psychiatric disorders, such as major depressive disorder (MDD), which occurs in 51–82% of PTSD cases [5–7]. PTSD heritability estimates range from 30 to 40% [8–10] and recent evidence suggests PTSD is highly polygenic [8, 11, 12].To date, only a few genomic loci have been implicated in the risk for PTSD. Recent large meta-analyses by the Million Veteran Program and the Psychiatric Genomics Consortium revealed ~ 100 significant loci for PTSD diagnosis or PTSD quantitative symptom traits [8, 11–14]. Transcriptomic studies using human postmortem prefrontal cortex (PFC) tissue have linked dysregulation of biological processes to PTSD, including GABA signaling, inflammation and cytokine effects, and glucocorticoid signaling [15–18].

Tandem mass spectrometry (MS/MS) has become an indispensable tool for obtaining unbiased, high-resolution proteomic data. Whole-proteome analysis is essential for understanding the molecular facets of the human brain because proteins and their changes provide unique insight into the state of the cell. The entire neuroproteome can only be profiled using mass spectrometry, which has comparable throughput and resolution of other functional genomic techniques. To fully understand the functional and system-level roles of central nervous system (CNS) cells in disease, quantitative investigation of the thousands of proteins expressed in multiple neuronal and non-neuronal cell types is essential. The roles of post-transcriptional modifications and the trafficking of transcripts and proteins must be integrated with other functional genomic data to better understand the dynamics of disease alterations to RNA and protein expression. Despite the potential value, few studies have been conducted in human postmortem brains of donors with major psychiatric illness and fewer still have sought to integrate this data with other genomic modalities. Additionally, as the final step of the central dogma of molecular biology, protein sequence and abundance may be the most relevant genomic level to identify potential therapeutic points of intervention.

Systems-level analyses of multi-omics datasets are essential tools for identifying molecular targets of disease processes beyond what coding gene expression alone could reveal [19]. Therefore, we performed proteomic tandem mass spectrometry on postmortem brains from individuals with PTSD, a psychiatric comparison group (MDD), and neurotypical controls and coupled this with genome-wide expression profiling of mRNA and miRNAs. We examined tissue from two prefrontal cortical regions: dorsolateral prefrontal cortex (DLPFC; Brodmann area 9/46) and subgenual prefrontal cortex (sgPFC; Brodmann area 25). These two regions were chosen based on previous clinical evidence of functional engagement in PTSD [20, 21]. We also developed a multi-modal bioinformatics analysis pipeline to link protein abundance with miRNA and RNA expression changes in postmortem brain.

We identified PTSD-specific protein differential expression signatures and co-expression patterns, including downregulation of interneuron-specific modules containing GABAergic proteins SLC32A1 and NEGR1. These protein modules also exhibited significant enrichment of risk genes for autism spectrum disorder (ASD) and major depressive disorder (MDD), suggesting shared molecular pathology and risk. In addition, we found that several miRNAs were upregulated in the PTSD brain, including *hsa-mir-589* and *hsa-mir-6786* and integrative analysis identified miRNAs enriched for disease-associated proteins and protein modules. Our findings highlight the use of large-scale multi-omics systems biology to unravel the effects of the neuroproteome in psychiatric disorders.

## Methods

### Human postmortem cohort

This study was conducted using frozen postmortem brain specimens from the University of Pittsburgh Medical Center. Individuals were a mix of European, Asian, and African American descent. Brain specimens were obtained during autopsies conducted at the Allegheny County Medical Examiner’s Office (Pittsburgh, PA, USA) after obtaining consent for donation from the next-of-kin. An independent committee of experienced clinicians confirmed the presence or absence of psychiatric illness for each subject using an expanded psychological autopsy approach [22]. The cohorts were matched for sex, age, PMI, and pH. A summary of sociodemographic and clinical details is listed in Additional file 1: Fig. S1 (the donors used in our proteomics experiments in Additional file 1: Fig. S1A, transcriptomics in Additional file 1: Fig. S1B and smRNA-seq in Additional file 1: Fig. S1C) and includes the presence of comorbid disorders, tobacco use, manner of death, and presence of drug and/or alcohol abuse. A total of 57 individuals (19 PTSD: 9 males, 10 females; 19 MDD: 9 males, 10 females and 19 healthy controls: 10 males, 9 females, see Additional file 1: Fig. S1A) were recruited by design for proteomics profiling and an expanded cohort of 66 individuals (22 PTSD: 11 males, 11 females; 22 MDD: 11 males, 11 females; and 22 healthy controls: 11 males, 11 females, see Additional file 1: Fig. S1B) for RNA-seq, and a subset of 57 individuals (17 PTSD: 8 males, 9 females; 22 MDD: 11 male, 11 female; and 18 healthy controls: 11 males, 7 females, see Additional file 1: Fig. S1C) for small RNA-seq Fresh frozen tissue samples (25 mg) of PFC from the sgPFC (BA 25) and the DLPFC (BA 9/46) were utilized for each donor.

Psychiatric history and demographic information were obtained via an extensive psychological autopsy including medical record collection and review; structured diagnostic interviews (SCID-5-RV and SCID-5-PD) with the next-of-kin or other knowledgeable informant; review of neuropathology and toxicology reports; and a postmortem diagnostic conference staffed by adult, child, and geriatric psychiatrists, clinical psychologists, and other senior psychiatric clinicians, that generates DSM-IV/5 and ICD-10-CM diagnoses (or their absence) for all subjects as previously described. Rates of depression were roughly equal between the cohorts with 47% comorbid for depression in the proteome cohort, and of the 22 individuals with PTSD in the transcriptome cohort, 50% were comorbid for depression.

### smRNA-seq library preparation

RNA was isolated from 20 mg of frozen postmortem brain tissue using a RNeasy Mini Kit with genomic DNA elimination, as described by the manufacturer (Qiagen). The RIN and concentration were assessed using a Bioanalyzer (Agilent). smRNA libraries were constructed using a QiaSeq miRNA library kit (Qiagen) from 1 µg of RNA. Samples were barcoded and sequenced on a HiSeq2500 (Illumina) at a read depth of 20M.

### Tissue collection and preparation for LC–MS/MS

Tissue was lysed in RIPA buffer with protease and phosphatase inhibitors (100 × halt inhibitor cocktail, Thermofisher) using a probe sonicator. Cellular debris was pelleted by centrifugation and the supernatant containing soluble proteins was collected. Soluble protein (20 µg in 10 µl) was aliquoted from the supernatant, additional water was added to a final volume of 100 µL. Samples were then added to 200 µL of ice-cold acetone and protein was precipitated overnight. The pellet was air dried and resuspended in 20 µL 8 M urea, containing 400 mM ammonium bicarbonate (pH 8) and reduced in dithiothreitol (DTT; 2 µL, 45 mM) at 37 °C for 30 min. Samples were alkylated with iodoacetamide (IAM; 2 µL, 100 mM) at room temperature (in the dark) for 30 min. Additional water was added and samples were enzymatically digested with sequencing-grade trypsin (1:40 trypsin:protein; Promega, Madison, WI, USA) at 37 °C for 16 h. The final volume was 80 µL. Digested proteins were acidified in 0.1% formic acid and desalted via column purification (C18 spin columns; The Nest Group, Inc; Southborough, MA, USA) and dried using a SpeedVac. Samples were stored in −80 °C until mass spectrometry analysis.

### Data-independent acquisition (DIA) mass spectrometry (MS)

Purified samples were resuspended in 0.2% trifluoroacetic acid (TFA)/2% acetonitrile (ACN) in water. DIA LC–MS/MS was carried out with a nano-ACQUITY UPLC system (Waters Corporation, Milford, MA, USA) connected to an Orbitrap Fusion Tribrid (ThermoFisher Scientific, San Jose, CA, USA) mass spectrometer. Samples were injected and loaded into a trapping column (nanoACQUITY UPLC Symmetry C18 Trap column, 180 µM × 20 mm) at 5 µL/min. Peptides were subsequently separated using a C18 column (nanoACQUITY column Peptide BEH C18, 75 µm × 250 mm). Mobile phases consisted of Mobile Phase A (0.1% formic acid in water) or Mobile Phase B (0.1% formic acid in ACN). Peptides were eluted with 6–35% gradient Mobile Phase B for 90 min and 85% Mobile Phase B for 15 min at 300 nL/min, 37 °C. All sample injections were interspersed by column regeneration and three blank injections. Data were acquired under data-independent acquisition (DIA) mode with an isolation window of 25 m/z. Full scan was in the 400–1000 m/z range, “Use Quadrupole Isolation” enabled at an Orbitrap resolution of 120,000 at 200 m/z and automatic gain control target value 4 × 10^5^. MS^2^ fragment ions were generated in C-trap with higher-energy collision dissociation at 28% and Orbitrap resolution of 60,000.

### Quality control for proteomics data

DIA spectra were searched against a *Homo sapiens* brain proteome fractionated spectral library generated from Data-Dependent Acquisition (DDA) LC MS/MS spectra (collected from the same Orbitrap Fusion mass spectrometer with HDC fragmentation) using Scaffold DIA software v. 1.2.1 (Proteome Software, Portland, OR, USA) [23]. Within Scaffold DIA, raw files were first converted to the mzML format using ProteoWizard v. 3.0.11748 [24]. The samples were then aligned by retention time and individually searched with a mass tolerance of 10 ppm and a fragment mass tolerance of 10 ppm. The data acquisition type was set to “Overlapping margins of 2 Da,” and the maximum missed cleavages were set to 2. Fixed modifications included carbamidomethylation of cysteine residues (+ 57.02). Dynamic modifications included phosphorylation of serine, threonine, and tyrosine (+ 79.96), deamination of asparagine and glutamine (+ 0.98), oxidation of methionine and proline (+ 15.99), and acetylation of lysine (+ 42.01). Peptides with charge states between 2 and 4 and 6–30 amino acids in length were considered for quantitation, and the resulting peptides were filtered by Percolator (v. 3.01) [25] at a threshold FDR of 0.01. Peptide quantification was performed by EncyclopeDIA (v. 0.9.2) [23], and six of the highest quality fragment ions were selected for quantitation. Proteins containing redundant peptides were grouped to satisfy the principles of parsimony, and proteins were filtered at a threshold of two peptides per protein and an FDR of 1%. Five DLPFC samples (3 CON, 1 MDD, and 1 PTSD) were excluded due to failed technical preparation.

Contribution of sociodemographic factors to the variation of protein abundance was assessed with principal component analysis (PCA) and variance partition analysis with R package *variancePartition* as previously described [26].

### Differentially expressed proteins

Differential expression analysis for proteins was performed with R package *limma* [27]. Proteins that were not registered UniProtKB/SwissProt [28] were filtered out. Any proteins with 0 peptides were also dropped. For each brain region, an empirical Bayes linear regression model was used to fit protein expression with PrimaryDx and covariates, including the age of death, ancestry, and sex. *P*-values were adjusted to control FDR.

### Rank-rank hypergeometric overlap analysis

A rank-rank hypergeometric overlap (RRHO) plot was made with R package *RRHO2* [29, 30]. For each pair of conditions, log_10_ fold changes of corresponding proteins in different conditions were compared after ranking.

### Quality control and data preprocessing for RNA-seq and smRNA-seq data

RNA-seq and smRNA-seq data in FASTQ files were mapped to the human reference genome and annotation GTF file (GRCh38, release 104) downloaded from ENSEMBL [31] with software *STAR* (v2.5.3a) [32] and counted with *featureCounts* (v1.5.3) [33].

For transcriptomics data, default mapping parameters were used and ENSEMBL annotations were applied. ENSEMBL identities were mapped with gene annotation using the *biomaRt* package in R [34]. Samples with overexpressed mitochondria genes that accounted for more than half of total counts were filtered out. Two PTSD sgPFC samples from the cohort were excluded from the following analysis.

For smRNA-seq, *STAR* parameter settings included *outFilterMultimapScoreRange* set to 0, *outFilterMatchNmin* set to 16, and *outFilterMatchNminOverLread* and *outFilterScoreMinOverLread* set to 0.3. In *featureCounts*, counting was conducted on fragments with exon annotations on the transcript level, stranded, and multiple assignments allowed. In total, 1365 miRNAs were found across all samples. Four DLPFC samples (1 CON, 1 MDD, and 2 PTSD) and 2 sgPFC samples (2 MDD) were excluded due to failed library preparation.

### Differentially expressed genes

Differential expression analysis for transcriptomics was performed with R package *DESeq2* [35] in a region-specific manner. In each region, genes with an average count less than 0.5 across all samples were filtered out. Then expression was modeled with PrimaryDx and covariates, including PMI (postmortem interval), sex, ancestry, the age of death, age square, RIN (RNA integrity number), and RIN square.

### Differentially expressed miRNAs

miRNA expression counts were extracted from smRNA-seq data. Differential expression analysis for miRNAs was performed with R package *DESeq2* [35] in a region-specific manner. In each region, miRNAs with an average count less than 0.5 across all samples were filtered out. Then expression was modeled with PrimaryDx and covariates, including the age of death, RIN (RNA integrity number), and sex.

### Pathway enrichment analysis

Pathway enrichment analysis of differentially expressed proteins (DEPs) was performed in QIAGEN IPA (QIAGEN Inc., https://digitalinsights.qiagen.com/ipa) [36]. A cutoff of *P*-value < 0.05 was used to define significantly changed pathways. Neuronal cell compartment pathway enrichment analysis of protein modules was conducted with SynGO (https://www.syngoportal.org/index.html) [37]. Threshold-free enrichment analysis was done using gene set enrichment analysis (GSEA) with R package *clusterProfiler* [38].

### Protein co-expression modules and network analysis

Protein co-expression modules were constructed with the R package *WGCNA* (Weighted Gene Co-expression Network Analysis) [39]. Control and MDD samples were used to construct MDD modules; control and PTSD samples were used to construct PTSD modules. The module-trait correlation was calculated between module eigengenes and sample traits, including PrimaryDx, age of death, PMI (postmortem interval), ancestry, and sex. Soft-threshold powers (6 for DLPFC conditions and 4 for sgPFC conditions) were used to achieve approximate scale-free topology in each condition. Protein modules were built with the *blockwiseModules* function in *WGCNA*. Modules were labeled with random colors. *P*-values were FDR-corrected to adjust for multiple comparisons. *TOMtype* was set to “signed” and *mergeCutHeight* was set to 0.1. Minimum of module sizes was set to 20.

Protein–protein interaction networks were inferred from mutual information with protein expression using algorithm ARACNE [40] implemented by R package *bnlearn*. The network organization of modules was visualized using the R package *igraph* [41]. Here each node represents a protein, and the lines between them show significant protein–protein co-expression. Finally, undirected key driver analysis was performed in the local networks of protein modules with R package *KDA* (https://labs.icahn.mssm.edu/binzhanglab/resources/) [42] to identify key driver proteins of the corresponding module.

### Cell type-specific enrichment analysis (CSEA)

R package *pSI* (http://genetics.wustl.edu/jdlab/psi_package/) [43] was used to perform cell type-specific enrichment analysis for each module (either MDD or PTSD). Cell type-specific gene expression profiles included neurons, astrocytes, microglia, oligodendrocytes, and endothelial cells, based on human data from Gene Expression Omnibus (GEO) with accession ID GSE73721 [44]. Gene expression was log-transformed, and mean values were calculated for each cell type. Cell type-specific enrichment for the *WGCNA* module was conducted with function *specificity.index* in the *pSI* package. Fisher’s exact test was used to test the significance with a pSI threshold of 0.05. The obtained *P*-values were FDR-corrected.

### Module preservation between MDD and PTSD

For each brain region and a given pair of MDD and PTSD modules, their module consistency was calculated using Fisher’s exact test to look for the overrepresentation of proteins of a paired module in the other. *P*-values were FDR-corrected, and module pairs were significantly preserved if FDR < 0.05. The enrichment score is represented by the odds ratio of protein overlap over the expected numbers.

### Comparison between transcriptomics and proteomics

The consistency of transcriptomic and proteomic changes was compared. For each brain region and disorder, genes with a *P*-value < 0.05 for disease association on both RNA and protein levels were selected. Log_2_ fold changes (log_2_(FC)) were compared for each gene. Transcriptomic log_2_(FC) were obtained from the previous transcriptomic study of MDD and PTSD from the same brain region [15].

### Protein-miRNA pairs

Correlations of protein-miRNA pairs were calculated based on protein and miRNA expression levels from the same individuals. Samples were selected with both proteomics and miRNA measurement. For control-PTSD, there were 27 matched samples in DLPFC and 31 in sgPFC; for control-MDD, there were 14 in DLPFC and 33 in sgPFC. miRNAs with average raw expression > 0.5 were kept and transformed to FPKM. A correlation test was performed for each protein and miRNA pair with protein log_10_(intensity) and miRNA log_2_(FPKM). A significant protein-miRNA connection is defined by *P*-value < 0.05.

### miRNA enrichment of DEPs and protein modules

miRNAs’ enrichment of DEPs and protein modules was estimated based on protein-miRNA pairs described above. For each miRNA, the enrichment score was the odds ratio of overrepresentation of DEPs (defined by protein-disease association *P*-value < 0.05) among all the proteins connected with the miRNA. Fisher’s exact test was applied to the enrichment to get a *P*-value. Significant DEP enrichment was defined by a *P*-value < 0.05.

miRNA-module enrichment was done similarly. For a given miRNA and a protein module, the enrichment score was the odds ratio of overrepresentation of the module membership among all the proteins connected with the miRNA. *P*-values were obtained from Fisher’s exact test. Significant enrichment of protein modules was defined as *P*-value < 0.05.

### Protein module enrichment of multi-trait TWAS results

GWAS summary statistics were downloaded from studies listed in Table 1. TWAS predictions for each trait were made with UTMOST [45] by joint analysis of individual predictions from all cortical, brain, and body tissues listed in Genotype-Tissue Expression (GTEx) project release v6p and v8 [46, 47]. UTMOST performs gene expression imputation across tissues and gene-level association tests to identify trait-associated genes from GWAS summary statistics. The complete list of 44 tissues is on the GTEx website (https://gtexportal.org/home/). The top 200 predicted genes were used for each trait to balance different psychiatric disorders. A Fisher exact test was performed for each protein module to test for the overrepresentation of top UTMOST predicted genes in the module among all genes. Significant enrichment was marked by a *P*-value < 0.05.

**Table 1:** List of GWASs of neurodegenerative and psychiatric diseases

Disease/disorders	Reference	Case	Control	N total	Loci identified	Ancestry group(s) and N of summary statistics used
Autism spectrum disorder (ASD)	Grove et al*.* 2019	18,381	27,969	46,350	12	All (EUR)
Alzheimer’s disease (ALZ)	Wightman et al. 2021	90,338	1,036,225	1,126,563	38	All (EUR/mixed)
Bipolar disorder (BIP)	Mullins et al. 2021	41,917	371,549	413,466	64	All (EUR)
Major depressive disorder (MDD)	Meng et al. 2024	88,316	902,757	991,073	53 novel	All (multi-ancestry)
Posttraumatic stress disorder (PTSD)	Nievergelt et al. 2024	150,760	1,130,173	1,280,933	95	EUR (*N* = 1,222,882)
Schizophrenia (SCZ)	Lam et al. 2019	56,418	78,818	135,236	19	EUR (*N* = 77,096)

*EUR* European ancestry

## Results

### Differentially expressed proteins in PTSD and MDD cases versus controls

**Fig. 1:**
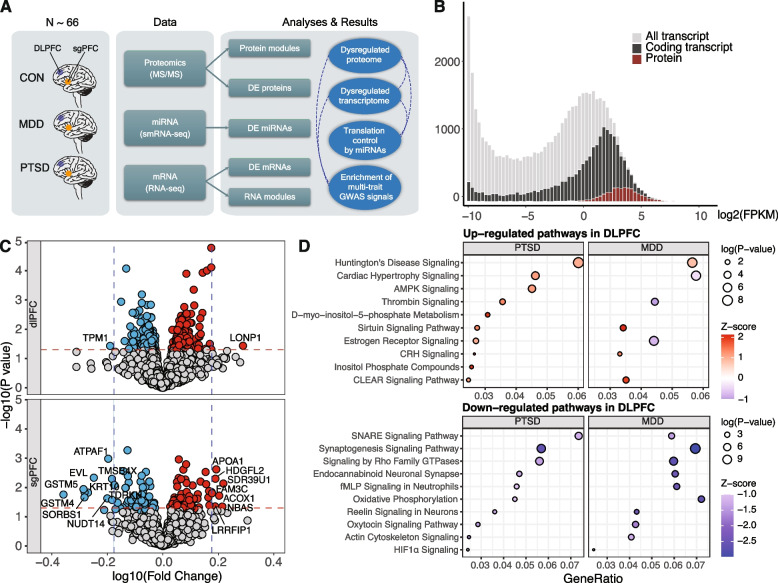
Multiomic data overview and differential expression analysis of proteins in PTSD. **A** A schematic overview of the study design and analysis with multiple -omics datasets employed in this study. Dashed lines indicate integration of data-inferred associations in disease. **B** Distribution of gene and protein expression (all transcripts; gray), protein-coding transcripts (black) and peptides (red). **C** Volcano plots showing DEPs in DLPFC (top) and sgPFC (bottom) of PTSD brains. Red (*P* < 0.05 and log fold change > 0) indicates upregulated proteins, and blue (*P* < 0.05 and log fold change < − 0.18) indicates downregulated proteins. **D** Top significantly enriched biological pathways in DLPFC and sgPFC for PTSD and MDD differentially expressed proteins

We analyzed postmortem brain samples from a large cohort (*N* = 57), including neurotypical control subjects (CON, *N* = 19), subjects with major depressive disorder (MDD, *N* = 19), and subjects with post-traumatic stress disorder (PTSD, *N* = 19). We obtained 109 samples in total for the two regions (DLPFC, *N* = 52, sgPFC, *N* = 57) after removal of technically failed library preparations (Fig. 1A,Additional file 1: Fig. S1A). We performed tandem mass spectrometry (LC MS/MS) on the samples to measure protein abundance levels using data-independent acquisition (DIA) [48] (Fig. 1A). After extensive and rigorous quality control of LC MS/MS (see “ Methods”), we performed differential expression analysis for 109 samples from 57 unique donors. Detailed sample information with corresponding demographics is listed in Additional file 1: Fig. S1A. We initially identified 2775 proteins and after rigorous quality control (see “ Methods”) 2598 remained. We compared protein to transcripts levels generated from a partially overlapping RNA-seq dataset [15]. As expected, proteins with higher corresponding RNA expression levels were preferentially captured over lower expressed mRNAs (Fig. 1B). We performed principal component analysis (PCA) to assess the effects of demographic confounders in protein expression variation. Regional differences in proteomic profiles were captured by PC1 (accounting for 29% of the variance) (Additional file 1: Fig. S1D). Previous characterization of the transcriptome of these donors identified significant differences between males and females [15]. Surprisingly, sex accounted for less of the expression variance (0.03%) at the proteomic levels using variance partitioning analysis [26] (Additional file 1: Fig. S1E).

Differential expression analysis identified disease- and brain region-specific proteins (*P*-value < 0.05, Fig. 1C). Overlap of differentially expressed proteins (DEPs) was moderate between MDD and PTSD from the same brain regions (28% DLPFC, 10.7% sgPFC) and was lower between different brain regions (5.3% and 2.8%) (Additional file 1: Fig. S2A). Rank-rank hypergeometric overlap (RRHO) [29, 30] showed convergent proteomic changes between MDD and PTSD from the same brain region but not across regions within the same diagnostic cohort (Additional file 1: Fig. S2B). In DLPFC, one protein, MACD1, was significantly upregulated in PTSD (FDR = 0.043). Eight proteins were significantly changed in MDD after controlling for multiple comparisons (FDR < 0.05, Additional file 2: Table S1), including GNB4, RAC1, and CNTFR (up) and LY6H, CNTN1, DPYSL4, LAP3, and SLC17A7 (down). MDD or PTSD protein levels were not significantly altered in the sgPFC at an FDR < 0.05 cut-off. Using a more liberal cutoff of *P*-value < 0.05, we identified 351 unique DEPs (DLPFC: 249, sgPFC: 112, 10 overlapping proteins) for PTSD. In MDD, we identified 361 unique DEPs (DLPFC: 243, sgPFC: 137, 19 proteins overlapping). A list of all DEPs and statistics across models appears in Additional file 2: Table S1.

We performed pathway enrichment analysis with Ingenuity Pathway Analysis (IPA) [36] using a ranked list sorted by (*P*-value < 0.05) to identify up- and downregulated biological functions. In DLPFC, we noted downregulation of pathways related to synaptic signaling (*P* = 4.90 × 10^−9^) and endocannabinoids (*P* = 8.51 × 10^−4^) indicating DEP enrichment in neuronal cell types, as well as upregulation of corticotropin-releasing hormone (CRH) signaling consistent with glucocorticoid dysfunction in PTSD [12] (Fig. 1D). In sgPFC, we found enrichment of several pathways including oxidative phosphorylation (*P* = 4.57 × 10^−4^) and Aryl hydrocarbon Receptor Signaling (*P* = 1.62 × 10^−3^). A full list of enriched pathways for PTSD is included in Additional file 3: Table S2.

### Proteomic analysis reveals unique molecular signatures and functions for PTSD compared to MDD

The genetic overlap between PTSD and MDD is high (approximately 70–80%) [49]; however, recent postmortem transcriptomic evidence suggests distinct molecular pathologies between the two disorders [15, 17]. Analysis of DEPs in MDD and PTSD postmortem brain tissue revealed moderate levels of overlap between the two diseases (Additional file 1: Fig. S3). In DLPFC, 243 DEPs for PTSD and 249 for MDD share an overlap of 107 DEPs (28% of all 385 psychiatric DEPs across disorders). While far fewer protein changes were observed in sgPFC, a similar trend was observed, with 19 overlapping DEPs (18% of combined) between the 69 DEPs for PTSD and 57 in MDD. Overall, top regulated DEPs for PTSD have significant or nominally significant changes in MDD, especially in DLPFC. Forty-one of the top 50 PTSD downregulated proteins (*P* < 0.05) nominally trend down (*P*-value < 0.1) in the MDD dataset. For top upregulated DEPs, a similar pattern was observed, with 32 of the top 50 upregulated PTSD proteins trending in MDD (*P*-value < 0.1). For these top 100 genes in PTSD, none of the MDD *t*-values carried an opposite sign.

Biological pathway enrichment results suggested shared and distinct molecular patterns between PTSD and MDD. MDD shared enrichment with PTSD for all the top 10 downregulated pathways, while only four of the top 10 upregulated pathways overlapped (Fig. 1D). Most converging pathway changes occurred in DLPFC (Additional file 1: Fig. S3). A full list of enriched pathways for MDD can also be found in Additional file 4: Table S2.

When PTSD was directly compared to MDD (not relative to a control group but to each other), we found many differences in the protein profiles (see Additional file 2: Table S1). Thirty proteins including GBB4 and MACD1 were significantly altered with MACD1 lower in MDD and GBB4 levels lower in PTSD. In DLPFC, pathway enrichment analysis of DEPs (*P-*value < 0.05) revealed dysregulation of glutamate biosynthesis (*P* = 2.52 $\times$ 10^−3^) as a top pathway (Additional file 3: Table S2). These data suggest there are differences in the proteomes of the PTSD and MDD cortex centering predominantly on vascular and neurotransmitter processing.

We compared the results from this study to a previously published PTSD multi-omics one [50]. We compared proteomic differential expression results from both brain regions of this study and that of mPFC from Daskalakis et al. and found correlation in global proteomic changes (*R*^*2*^ ranging from 0.005 to 0.05, *P*-values ranging from 5.6 × 10^−4^ to 2.2 × 10^−16^) that were moderate but significant (Additional file 1: Fig. S4). We also found a number of differentially expressed proteins (DEPs) and pathways that overlapped. We found 105 overlapped DEPs between the two studies (see Additional file 4: Table S3), including 65 overlapped proteins in PTSD and 40 proteins in MDD. The overlapped PTSD DEPs include downregulated GABAergic genes SLC32A1, NEGR1, and PACSIN1 (see Additional file 1: Fig. S4E,F), which we consider are high-confidence PTSD DEPs, especially NEGR1 which is also a risk gene for multiple psychiatric disorders including MDD [51] and SCZ [52]. The 40 overlapped MDD DEPs also include SLC32A1 and NEGR1, along with the interneuron marker gene GAD2, indicating that the two studies had consistent findings and corroborate our observation of decreased GABAergic proteins in the frontal cortex of PTSD and MDD brains.

### Protein co-expression analysis reveals disease-specific modules and evidence for presynaptic alterations and interneuron dysfunction in PTSD and MDD

**Fig. 2:**
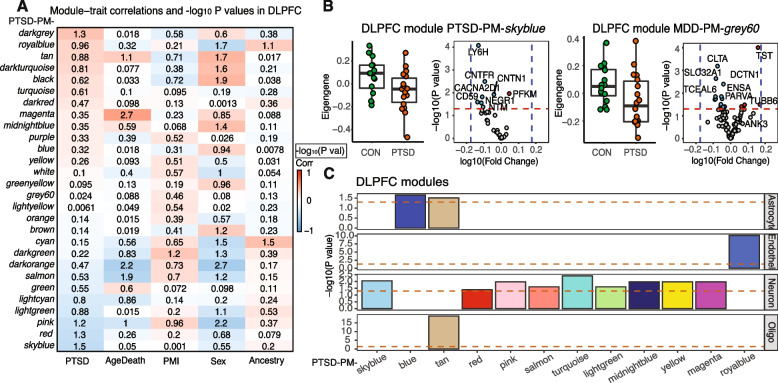
Network Co-expression analysis of proteomics data. **A** Module-trait correlation between protein expression correlations in the DLPFC of PTSD brains and demographic features (PrimaryDx, age of death, PMI, sex, ancestry). Color in each cell reflects correlation between module eigenprotein and PrimaryDx and the number represents significance (− log_10_(*P*-value)) of that correlation. Module names are abbreviated as color codes only. **B** Boxplots of eigenproteins and volcano plots of differential expression patterns of the proteins in PTSD-associated modules PTSD-PM-*skyblue* (left) and *red* (right) (vertical dash lines indicate log_10_(fold change) = ± 0.18. Module PTSD-PM-*skyblue*, *P* = 0.03; module PTSD-PM-*red*, *P* = 0.04. *P* indicates eigenprotein change significance between PTSD and CON. **C** Cell type enrichment analysis showing the enrichment of cell type markers in protein modules. Brown dashed line indicates significantly enriched markers, *P* < 0.05

In addition to identifying disease-associated protein signatures, we also sought to understand the molecular organization of the PTSD proteome by examining whether and to what extent proteins co-expressed with each other. We applied *WGCNA* [39] to find modules based on protein co-expression across donors and diagnostic cohorts (Fig. 2A). Twenty-five modules were identified for MDD and 28 for PTSD in DLPFC. Modules PTSD-PM (Protein Module)-*skyblue* (correlation $r$ = − 0.37) and PTSD-PM-*red* (correlation $r$ = 0.34) were significantly associated with PTSD diagnosis (Fig. 2B) (FDR < 0.05), and modules MDD-PM-*grey60* (correlation $r$ = − 0.42) and MDD-PM-*darkred* (correlation $r$ = − 0.36) were significantly associated with MDD (FDR < 0.05) (Additional file 1: Fig. S5). PTSD protein module PTSD-PM-*skyblue* is mainly composed of downregulated proteins, including LY6H, CACNA2D1, CD59, CNTN1, NTM, NEGR1, and OPCML. *OPCML* has been identified as a risk gene for PTSD [12] and *NTM* is a risk gene for autism [53] (Fig. 2B). NEGR1, NTM, and CACNA2D1 are hub proteins in both PTSD module PTSD-PM-*skyblue* and MDD module MDD-PM-*grey60* (Additional file 1: Fig. S6). Protein module information is included in Additional file 5: Table S4.

We next used cell type-specific enrichment analyses (CSEA) [54] to identify cell types associated with functionally distinct protein modules. CSEA uses a cell type-specific reference profile generated with translating ribosomal affinity purification (TRAP) from transgenic BACarray reporter mice. Nine PTSD protein modules were found to be enriched for neuronal markers, including modules PTSD-PM-*skyblue* and PTSD-PM-*red* in DLPFC (Fig. 2C). We also observed enrichment of cell type markers for astrocytes (2 modules), oligodendrocytes (1 module), and endothelial cells (1 module) (Fig. 2C).

Because protein function is largely based on its location in the cell, we hypothesize that PTSD DEPs would localize to common compartments of the cell. Synaptic compartment enrichment analysis with synGO [37] found that modules PTSD-PM-*skyblue* and MDD-PM-*grey60* were enriched for proteins localized to the presynaptic compartment (Additional file 1: Fig. S7 and Additional file 6: Table S5). The PTSD-associated protein module, PTSD-PM-*red*, includes GABAergic interneuron proteins including SLC32A1 and PACSIN1, which are also members of MDD module MDD-PM-*darkred* (Additional file 1: Fig. S6). SLC32A1 encodes solute carrier family 32 member 1, a GABA/glycine vesicular transporter. Decreased protein levels of SLC32A1 is consistent with previously reported decreases in its transcript levels in PTSD frontal cortex [15]. PACSIN1 (Protein Kinase C and Casein Kinase Substrate in Neurons 1 or syndapin) plays a key role in regulating synaptic development in hippocampal interneurons [55, 56], and mediating the modulatory effect of antipsychotic drugs in response to N-methyl-D-aspartate (NMDA) and glutamate [57]. Pathway analysis identified modules PTSD-PM-*red* and MDD-PM-*darkred* were also enriched for proteins in the presynaptic compartment (adjusted *P-*value 1.54 × 10^−6^ and 5.94 × 10^−5^, respectively) (Additional file 1: Fig. S7C,D).

### Differentially expressed transcripts and co-expressed gene modules in PTSD and MDD

In parallel with our proteomics characterization, we performed RNA sequencing on the same brain regions and donors from this cohort to profile their transcriptomic changes. After rigorous quality control of these RNA-seq samples, we removed 2 samples from sgPFC and performed differential expression analysis for 130 samples from 66 donors (DLPFC *N* = 66, sgPFC *N* = 64, see Additional file 1: Fig. S1B). PCA revealed that sex and brain region correlated with top principal components (Additional file 1: Fig. S8A,B) and accounted for most variance among all demographic factors (Additional file 1: Fig. S8A-D), which is consistent with previous characterizations that PTSD’s impact on brain transcriptomes differs by brain region and sex [15]. PCs 1 and 2 accounted for 41% and 14% of the variance in transcript expression.

**Fig. 3:**
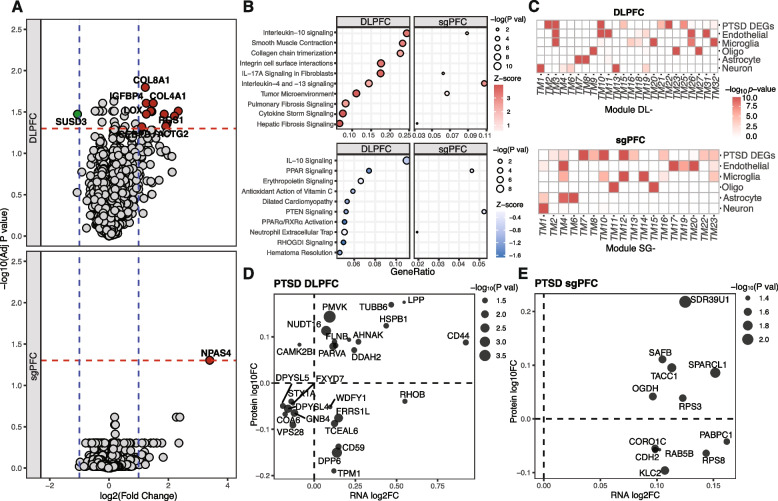
Gene expression and transcriptome network analysis of PTSD cortical regions. **A** Volcano plots showing DEGs in DLPFC (left) and sgPFC (right) of PTSD cortical regions. Red (*P *< 0.05) indicate upregulated genes and blue (*P *< 0.05) indicate downregulated ones. Red lines indicate *P*-value = 0.05 and black lines indicate |log fold change| > 0.18. **B** Pathway enrichment analysis of differentially expressed genes in PTSD and MDD DLPFC and their overlap with sgPFC. **C** Enrichment of DEGs and cell types in gene modules constructed by *WGCNA* for DLPFC (top) and sgPFC (bottom). Color indicates significance of enrichment (−log_10_(*P*-value)). Module names are abbreviated as indices only. Comparison of transcriptional and protein differential expression are shown in PTSD DLPFC (**D**) and sgPFC (**E**))

Gene expression analysis of the transcriptome identified 30 differentially expressed genes (DEGs) in DLPFC and only one gene in sgPFC that were significantly changed between PTSD and CON groups (FDR < 0.05, see Fig. 3A). The most changed genes in PTSD were *LINS1* (downregulated, adjusted *P-*value = 4.85 × 10^−5^, log_2_FC = − 0.44) in DLPFC, and *NPAS4* (upregulated, adjusted *P-*value = 0.0496, log_2_FC = 3.40) in sgPFC. No DEGs were shared between the regions; however, in both regions, there were more upregulated DEGs (70% and 100% in DLPFC and sgPFC, respectively) than downregulated ones. Gene set enrichment analysis of DEGs found enrichment of immune-related pathways in both brain regions, with moderate sharing of top dysregulated pathways between the two regions (Fig. 3B). Cell type-specific enrichment analysis (CSEA) [38] of DEGs pointed to enrichment of transcripts in microglia and endothelial cells (Fig. 3B).

To better understand the transcriptomic organization of these cortical regions in PTSD, we performed *WGCNA* [39] to identify co-expression gene modules. We found 42 RNA co-expression modules in DLPFC and 25 in sgPFC (Fig. 3C, Additional file 1: Fig. S9). Among DLPFC modules, seven were positively and six were negatively associated with PTSD, while in sgPFC, two were positively and five were negatively associated. Of note, among PTSD-associated modules, two (DL(DLPFC)-*TM*(transcriptome module)*1* and DL-*TM13*) were enriched for neuronal cell markers and one (SG(sgPFC)-*TM4*) was enriched for microglia markers (Fig. 3C, Additional file 1: Fig. S9). In both brain regions, most PTSD- and MDD-DEGs exhibited consistent changes (on average 58 and 55%, respectively) in direction and fold-change across both transcript and protein levels (Fig. 3D,E and Additional file 1: Fig. S10).

Comparison between RNA and protein coexpression modules showed high preservation levels between the two modalities. The majority of protein modules significantly overlap with RNA modules: 18 out of 29 protein modules in DLPFC and 5 out of 7 protein modules in sgPFC (Additional file 1: Fig. S11 and Additional file 5: Table S4), exhibiting a similar level of coexpression module preservation between RNA and protein as other published multiomics studies [58].

### Differentially expressed miRNAs in PTSD and MDD

**Fig. 4:**
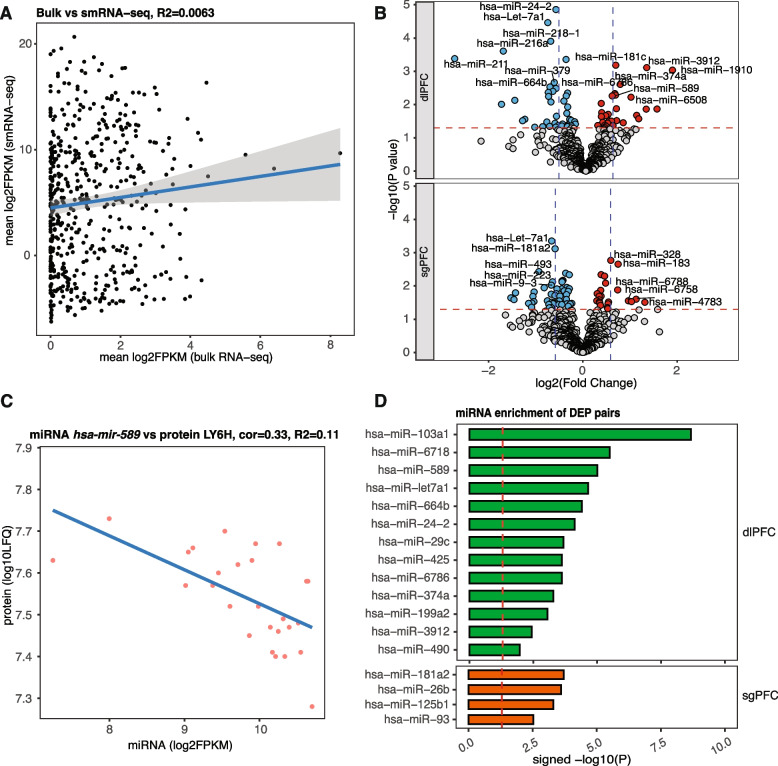
miRNA dysregulation in PTSD and its effect on the proteome. **A** Comparison of miRNA abundance captured using bulk mRNA-seq and smRNA-seq results. **B** Volcano plots showing differential expression patterns of miRNAs in PTSD DLPFC and sgPFC regions. Dashed lines: log_2_(fold change) = ± 0.5 and* P*-value = 0.05. **C** Examples of correlations between miRNA MIR589 and disease-associated protein LY6H. **D** miRNA enrichment analysis of DEPs to identify regulatory miRNAs and their targets. Bar heights (signed − log*P*) indicate significances of enrichment

We investigated the upstream, posttranscriptional mechanisms linking proteomic changes with transcript changes in the brain, by measuring the abundance of miRNAs for the same cohort. We generated RNA sequencing libraries enriched for small non-coding RNAs (smRNA-seq) from 53 samples of DLPFC and 55 samples from sgPFC (Additional file 1: Fig. S1C). We found notable differences in both coverage and total expression levels of all ncRNAs between bulk RNA-seq of total RNAs and small RNA-seq (Additional file 1: Fig. S12A). A total of 1365 miRNAs passed quality control, mapping, and filtering criteria for both brain regions. smRNA-seq provided higher sequencing coverage and deeper depth of shorter transcripts, especially miRNAs, than those from total RNAs, irrespective of region and diagnosis (see Fig. 4A). Supervised hierarchical clustering analysis [39] of these miRNA samples revealed stronger local clustering patterns for primary diagnoses (PrimaryDx) and brain region than other demographic features (e.g., sex, age at death, ancestry) and technical sequencing variables (RNA integrity number (RIN), postmortem interval (PMI)) (Additional file 1: Fig. S12B), consistent with PCA showing high correlation of brain region with top PCs (Additional file 1: Fig. S12C). Next, we performed differential expression analysis of miRNAs to identify changes in PTSD cases vs. controls. We identified seven miRNAs (six in DLPFC including *hsa-mir-24–2*, *hsa-let-7a1*, *hsa-mir-218–1*, *hsa-mir-216a*, *hsa-mir-211*, and *hsa-mir-425* and two in sgPFC including *hsa-let-7a1* and *hsa-mir-181a2*) surviving stringent correction for multiple testing. We identified 21 differentially expressed miRNAs in MDD. Three miRNAs (*hsa-let-7a1*, *hsa-mir-24–2*, *hsa-mir-181a2*) overlapped with PTSD in direction and magnitude (Fig. 4B, Additional file 7: Table S6). Analysis of differential miRNA expression revealed brain region-specific changes (Additional file 1: Fig. S13A). Gene set enrichment analysis (GSEA) [38] of differentially expressed miRNAs identified more suppressed (downregulated) pathways than activated (upregulated) ones for PTSD in both DLPFC and sgPFC, including suppression of miRNAs regulating cardiac muscle hypertrophy (Additional file 1: Fig. S14 and Additional file 8: Table S7). It is interesting to note that cardiac hypertrophy signaling was a regulated pathway in PTSD and MDD (Fig. 1D, Additional file 3: Table S2). We found specific decreases in RAC1 and RHOB protein and increases in RHOT1 protein which are among the targets of *hsa-mir-19a*, *hsa-mir-19B*, and *hsa-mir-17* and are all downregulated further suggesting PTSD directed changes to protein expression through miRNA mechanisms.

To investigate whether there are miRNA co-expression patterns, we performed *WGCNA* on our miRNA dataset (Additional file 1: Fig. S13B). We identified six miRNA co-expression modules with significant trait relationships (Additional file 1: Fig. S13B). One miRNA co-expression module, MiM(miRNA module)-*turquoise*, had significant association with PTSD (Additional file 1: Fig. S13C), indicating the enrichment of PTSD-associated miRNAs in this module. We identified 18 up- and seven downregulated miRNAs within module MiM-*turquois*e with a *P*-value < 0.05 (Additional file 1: Fig. S13D). Among the most significantly regulated miRNAs was *hsa-mir-589* (upregulated 1.56-fold in DLPFC), which has previously been implicated in MDD pathophysiology [59].

### miRNA integration with PTSD DEPs prioritizes hsa-mir-589 and hsa-mir-6786

Because miRNAs are known to regulate protein translation (normally through inhibition), we hypothesized that protein-mRNA pairs with fold changes in opposite directions in PTSD (Fig. 3D,E) might be regulated by disease-associated miRNAs. We found that 12 out of 15 DLPFC DEPs (ACADVL, CACNA2D1, CD59, CYB5B, DPP6, EXOC7, FRRS1L, RAC1, RHOB, TPM3, WDFY1, YWHAE) and five out of nine sgPFC DEPs (APOA1, NDRG2, PABPC1, RAB5B, RPS8) were associated with both expression and target 3′-UTR sequences of PTSD regulated miRNAs (Additional file 9: Table S8). To better understand the relationship between protein expression and miRNA function we integrated these two datasets. First, we performed Pearson’s correlation test between the normalized expression levels of the protein (in log_10_LFQ intensity) and the miRNA (in log_2_FPKM). An example of miRNA to protein correlation is provided in Fig. 4C for proteins LY6H with miRNA *hsa-mir-589*. Second, for each miRNA, we calculated an enrichment score for DEPs among all the proteins associated with the miRNA using Fisher’s exact test. Using this approach, we identified miRNA changes that were PTSD- or MDD-specific or associated with both disorders (Fig. 4D, Additional file 1: Fig. S15).

In DLPFC, 21 miRNAs had enrichment for PTSD DEPs (Fig. 4D), MDD DEPs (Additional file 1: Fig. S15B), or both. In the sgPFC, four miRNAs had enrichment for PTSD DEPs (Additional file 1: Fig. S15C). The miRNA with the most significant enrichment (*P* = 2.15 $\times$ 10^−9^) for PTSD-specific DEPs was *hsa-mir-103a1*, which was previously shown to be downregulated in blood plasma of patients with childhood traumatization [60]. The miRNAs that were most significantly enriched for both MDD and PTSD DEPs included *hsa-mir-6786* (*P* = 3.22 $\times$ 10^−6^ (PTSD) and 1.38 $\times$ 10^−11^ (MDD)) and *hsa-mir-589* (*P* = 9.85 $\times$ 10^−6^ (PTSD) and 3.95 $\times$ 10^−9^ (MDD)). While little is known about the function of *hsa-mir-6786*, *hsa-mir-589* has been previously implicated in depression [61] and was a member of the PTSD-associated miRNA module MiM-*turquoise* (Additional file 1: Fig. S13D). In addition, one of its predicted targets from TargetScan is the GABA vesicular transporter protein SLC32A1, which is significantly downregulated in PTSD DLPFC. We identified nine miRNAs exhibiting significant enrichment for associated DEPs (nominally significance of *P* < 0.05) in sgPFC (Fig. 4D). *hsa-mir-181a2* was the most significantly enriched miRNA for PTSD DEPs (*P-*value = 1.94 $\times$ 10^−4^), while *hsa-mir-24–2* (*P-*value = 2.81 $\times$ 10^−5^) and *hsa-mir-9–3* (*P*-value = 3.41 $\times$ 10^−5^) had the most significant enrichment MDD-specific DEPs. In addition, *hsa-mir-125b1* was enriched for both MDD and PTSD DEPs (*P-*values are 3.44 $\times$ 10^−3^ and 4.90 $\times$ 10^−4^, respectively). Taken together, these findings suggest direct pathological changes in protein levels by individual miRNAs specific to PTSD cortical regions.

### Integrative analysis identifies converging regulatory mechanisms in PTSD protein organization

**Fig. 5:**
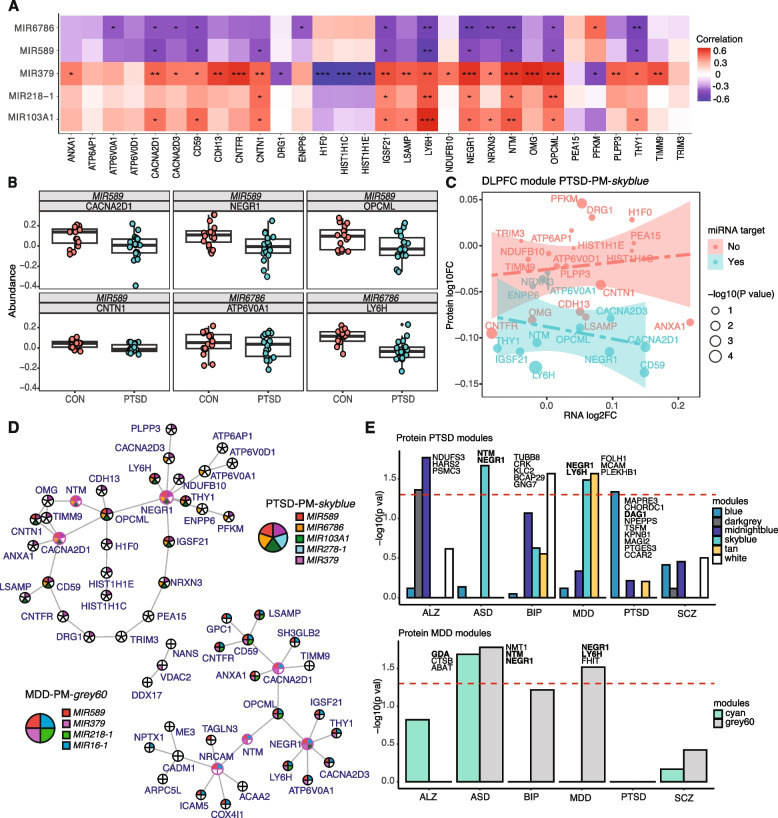
miRNA-regulated disease-associated protein modules. **A** Correlations between miRNAs with PTSD-associated protein module PTSD-PM-*skyblue*. Colors in heatmap indicate the correlation levels between miRNAs and proteins. miRNA-protein pairs with *P*-value < 0.05 are plotted. **B** Disease-specific protein abundances (log_10_ Intensity) of *hsa-mir-589* targets (CACNA2D1, NEGR1, OPCML, CNTN1), and *hsa-mir-6786* targets (ATP6V0A1, LY6H) in module PTSD-PM-*skyblue. ***C** Comparison of transcriptional and protein differential expression in PTSD module PTSD-PM-*skyblue* and grouped linear regression by proteins association with miRNAs. Red, proteins without miRNA association; blue, proteins with miRNA association. **D** Key driver analysis (KDA) plot of PTSD protein module PTSD-PM-*skyblue* (top) and MDD protein module MDD-PM-*grey60* (bottom). Pink circles indicate key drivers. Colors of individual slices refer to putative miRNA regulation of the protein. **E** Top: enrichment of GWAS risk genes from TWAS analysis in the proteomic modules of PTSD (top) and MDD (bottom), including Alzheimer’s disease (ALZ), autism spectrum disorder (ASD), bipolar disorder (BIP), major depressive disorder (MDD), post-traumatic stress disorder (PTSD) and schizophrenia (SCZ). Bolded proteins are DEPs (*P*-value < 0.05) in DLPFC. Colors of bars correspond to their module names. miRNA name abbreviations: *MIR103A1, hsa-mir-103a1*; *MIR218 -1, hsa-mir-218–1*; *MIR379, hsa-mir- 379*; *MIR589, has-mir-589*; *MIR6786, hsa-mir-6786*. PM- indicates protein module

While our initial analysis suggested individual miRNA to protein dysregulation is occurring, we hypothesized that miRNAs may be regulating groups of proteins in a coordinated fashion. Therefore, we sought to identify whether specific miRNAs regulate PTSD proteomic co-expression modules by using Fisher’s exact test to identify enrichment of miRNA-associated proteins within each protein module. A significant enrichment score (*P*-value < 0.05) indicated overrepresentation of protein targets of a particular miRNA within each protein module, and thus a higher likelihood of regulation of the network by the miRNA. We found five differentially expressed miRNAs were significantly (*P* < 0.05) correlated with protein abundance in the PTSD-associated module PTSD-PM-*skyblue* (Fig. 5A). *hsa-mir-6786* and *hsa-mir-589* were correlated (*P*-value < 0.05) with approximately 50% of proteins (13/30) in module PTSD-PM-*skyblue* and a majority of these pairs were negatively correlated (21 out of 22). We compared our miRNA-protein pairs with those predicted by TargetScan [62, 63] and found that the DEPs LY6H and ATP6V0A1 are targets of *hsa-mir-6786*. TargetScan also identified six proteins present in *skyblue* that were negatively associated with *hsa-mir-589* expression, including CACNA2D1, CNTN1, THY1, OPCML, CD59, and NEGR1 (Fig. 5A,B, Additional file10: Table S9). Because miRNAs generally act to reduce translation of proteins, we predicted negative correlations between miRNAs and their regulated protein targets. Both CACNA2D1 and CNTN1 were also confirmed in an independent miRNA database MirBase [64, 65] as targets of *hsa-mir-589*.

Shared and unique miRNAs were enriched for MDD modules as well. In module MDD-PM-*grey60*, six miRNAs were significantly enriched (*P*-value < 0.05), and three were negatively correlated with expression of eight out of nine pairs of the proteins in module MDD-PM-*grey60*, including *hsa-mir-6786*, *hsa-mir-589*, and *hsa-mir-4745* (Additional file 1: Fig. S16A). Both *hsa-mir-6786* and *hsa-mir-589* were shared with PTSD. Similar to module PTSD-PM-*skyblue*, module MDD-PM-*grey60* included predicted targets of *hsa-mir-6786*, including ATP6V0A1, GPC1, ICAM5, and LY6H, and those of *hsa-mir-589*, including CACNA2D1, NEGR1, OPCML, CD59, LSAMP, and SH3GLB2. The regulatory pairs of *hsa-mir-589*-CACNA2D1 and *hsa-mir-6786*-ICAM5 were also confirmed by MirBase. Module MDD-PM-*grey60* also included the predicted targets of *hsa-mir-4745*, including CNTFR, CD59, NEGR1, and SH3GLB2. In the module MDD-PM-*darkred*, only *hsa-mir-4745* was found to be enriched and negatively correlated with 8 associated proteins (Additional file 10: Table S9). This module contained predicted targets of *hsa-mir-4745*, including GPRIN1, PACSIN1, and MAPT (Additional file 1: Fig. S6). Among these, the regulation of PACSIN1 by *hsa-mir-4745* was confirmed in MirBase. The full miRNA-module enrichment results are annotated in Additional file1: Fig. S16B.

We further investigated if miRNAs could be contributing to the divergence between differential abundance changes of transcripts and proteins. Groupwise regression analysis showed opposite correlations (*r*_*miRNA*_ = − 0.14 and *r*_*no miRNA*_ = 0.027) between transcript and protein changes in proteins with or without predicted miRNA regulation (Fig. 5C). We performed this analysis on module MDD-PM-*grey60* and miRNA-targeted proteins showed similar negative correlations in contrast with non-target proteins (Additional file 1: Fig. S17). Key driver analysis [42] confirmed our hypothesis that proteins targeted by putative regulating miRNAs are closely connected to hub proteins NEGR1, CACNA2D1, and NTM (9 of 11 targets of *hsa-miR-589* and *hsa-miR-6786* are directly connected) in module PTSD-PM-*skyblue*. Similar patterns were observed in module MDD-PM-*grey60* (Fig. 5D). Taken together, these analyses identify specific miRNA-protein pairs that likely play significant roles in PTSD pathophysiology by controlling protein abundances across coordinated proteomic networks.

### Integrative analysis identified pan-psychiatric genetic risks in PTSD protein modules

Finally, we tested for enrichment of genetic risk signals for other neurological and neuropsychiatric disorders in protein module PTSD-PM-*skyblue* and protein module MDD-PM-*grey60* (Fig. 5E). Several studies have identified high genetic and transcriptomic overlap among these disorders, thus we reasoned this was likely [15, 66]. We included the latest GWAS results for Alzheimer’s disease (ALZ) [67], autism spectrum disorder (ASD) [53], bipolar disorder (BIP) [68], schizophrenia (SCZ) [69], MDD [70], and PTSD [14]. Specific details of each GWAS summary statistics are listed in Table 1. We used UTMOST (unified test for molecular signatures) [45], a cross-tissue transcriptome-wide association algorithm based on genotype-tissue expression (GTEx) v6p [46], to integrate gene expression across all brain tissues to infer disease-specific genes implicated from GWAS summary statistics of each disease. For each protein module in PTSD and MDD (Fig. 5E), we calculated an enrichment score for the top UTMOST-inferred genes with Fisher’s exact test. PTSD module PTSD-PM-*blue* was enriched for PTSD risk signals. Interestingly, we found the PTSD module PTSD-PM-*skyblue* was enriched for both ASD (*P*-value = 0.022) and MDD (*P*-value = 0.033) (Fig. 5E, top), containing transcriptome-wide associated genes including DEPs NEGR1, and LY6H for MDD and NTM and NEGR1 for ASD. We also identified one protein module (PTSD-PM-*midnightblue*) with highly significant enrichment (*P*-value = 0.017) for ALZ risk variants. We found evidence for enrichment of psychiatric risk genes in MDD modules as well (Fig. 5E, bottom). We found MDD module MDD-PM-*grey60* is enriched for MDD risks (*P*-value = 0.030) and similar to PTSD, module MDD-PM-*grey60* was also enriched for ASD risk (*P*-value = 0.017). Module enrichment results from joint analysis of all tissues also found enrichment of disease-associated modules in multiple psychiatric traits, including module PTSD-PM-*skyblue* and MDD-PM-*grey60* enrichment for ASD (*P*-value = 0.046 and 0.009, respectively) and BIP (*P*-value = 0.035 and 0.041, respectively), and MDD module MDD-PM-*darkred* enriched for SCZ (*P*-value = 0.010) (Additional file 1: Fig. S18A,B). To take advantage of the latest release of GTEx, we updated the UTMOST package and performed both joint analysis of brain tissue and cortical region TWAS using GTEx release v8. We found no additional enrichment of psychiatric disorder risk genes within our joint brain region analysis (Additional file 1: Fig. S18C,D) or within our joint cortical region analysis (Additional file 1: Fig. S18E,F). However, we did find significant risk for MDD enrichment in MDD-PM-*darkred* and identified DEPs AKAP5, ACTN4, PACSIN1, and MYH14. Taken together these data suggest that risk signals are harbored within dysregulated proteomic networks of PTSD and MDD and suggest a possible mechanism by which patients with stress disorders are at greater risk for developing other neuropsychiatric disorders.

## Discussion

In this study, we present a comprehensive, multi-omic evaluation of the neuroproteome of the human prefrontal cortex across neurotypical controls, MDD, and PTSD donors. Our investigation centered on two frontal cortical regions implicated in stress disorders: the DLPFC and sgPFC. This is one of the largest postmortem case/control proteomics cohorts for PTSD. It shares the findings of a number of differentially expressed proteins and enriched pathways from other large PTSD multiomics studies of other cortical regions but also provides unique insight of additional genomic mechanisms of miRNAs regulating the proteome in PTSD and MDD brain. By integrating protein, mRNA, and miRNA profiles from the same donors, we deeply characterize the mechanisms that control PTSD and MDD-related alterations on protein levels associated with the pathophysiology of these disorders. In addition, we identify dysregulated protein networks harboring risk signals for the development of other psychiatric disorders.

One model of PTSD neurobiology suggests top-down impairment of the fear circuitry of the brain [71]. Decreased prefrontal cortical activity has been observed in PTSD patients [72–76] and this likely results in the hyper-activation of subcortical regions (e.g., amygdala) resulting in exaggerated responses to fearful stimuli, a hallmark of PTSD. We have also identified the PTSD-associated protein module PTSD-PM-*red* that includes the downregulated interneuron protein SLC32A1, a vesicular GABA transporter that loads GABA neurotransmitter into synaptic vesicles. Further, *SLC32A1* transcript is a putative target of *hsa-mir-589* (Additional file 7: Table S6). We previously found that *SLC32A1* transcript was closely connected to expression of other interneuron molecular key drivers in PTSD postmortem brain tissues and was downregulated in the DLPFC. Both the PTSD-PM-*skyblue* and PTSD-PM-*red*, and the MDD-PM-*grey60* and MDD-PM-*darkred*, are also significantly enriched for proteins specific to the presynaptic compartment (Additional file 1: Fig. S7 and Additional file 6: Table S5) including LY6H, which regulates neurotransmitter trafficking [77]. *hsa-mir-589* expression is negatively correlated with several proteins in these modules including CACNA2D1, CACNA2D3, NEGR1, and OPCML and regulates expression of the GABA transporter SLC32A1 which is significantly downregulated in PTSD. We hypothesize that PTSD-associated miRNA, *hsa-mir- 589*, acts through its effector protein network (protein module PTSD-PM-*skyblue*), which is likely involved in neuronal plasticity and interneuron function, to disrupt synaptic transmission in the prefrontal cortex to other fear centers of the brain. Taken together these findings point to impaired GABAergic function in the cortex of PTSD patients and represent one possible molecular mechanism by which prefrontal activity inhibition manifests in PTSD.

Significant progress has been made to identify cross-disorder overlap in the genomic signals among psychiatric disorders. Recently, Gandal et al. [66] identified significant correlations in the transcriptomes of prefrontal cortical regions among schizophrenia (SCZ), autism spectrum disorder (ASD), and bipolar disorder (BIP) and some overlap with MDD and alcohol use disorder. In our previous work [15], we compared the DLPFC transcriptome of PTSD to these same disorders and after uniform processing of our data, identified significant transcriptomic correlation between PTSD versus SCZ, ASD and BIP. However, those findings did not identify significant correlation between PTSD and MDD. In this study, we likewise found only moderate overlap in DEPs and regulated miRNAs between the two disorders but there was some convergence in protein co-expression patterns. Both the PTSD module PTSD-PM-*skyblue* and MDD module MDD-PM-*grey60* are also enriched for ASD and MDD GWAS signals (Fig. 5E). The PTSD-associated neuronal module, PTSD-PM-*skyblue*, contains 30 proteins, including downregulated DEPs, CACNA2D1, OPCML, and NEGR1, which are shared by the MDD-associated neuronal module MDD-PM-*grey60*. OPCML is an opioid receptor that has been previously reported as a PTSD risk gene [12]. OPCML has also been shown to be associated with susceptibility for ASD, SCZ, and MDD [78–80]. NEGR1 regulates neural growth [81], connectivity, and cognitive functions [82] and has been reported as a genetic risk gene for MDD [83–86] and other comorbid disorders such as anxiety [87] and autism [82]. CACNA2D1 encodes the ${\alpha }_{2}\delta$−1 subunit of the voltage-gated calcium channel and affects multiple brain disorders including ASD and SCZ [88]. These findings point to common disruption of cell adhesion and neural connectivity processes among PTSD, MDD, and multiple other psychiatric disorders.

Multi-omic profiling of the same cohort of donors has enabled us to uncover the mechanisms by which PTSD and MDD affect the brain, providing novel insights into the alterations of molecular regulatory events in the stressed brain. In our study, we found links between upregulated miRNAs and targeted groups of decreased proteins, while transcripts levels remain unchanged, pointing to aberrant post-transcriptional regulations in the stressed brains. For example, miRNA *has-mir-589* shows negative associations with 30% of protein members of PTSD module PTSD-PM-*skyblue*, and its key drivers are predicted targets from miRNA databases (Fig. 5B), pointing to a targeted disruption of synaptic protein expression in GABAergic interneurons due to miRNA dysregulation. We also compared our study to a recent large multi-omics study of the PTSD and MDD mPFC [50]. We found high correlation in global proteomic abundance and moderate overlap in PTSD and MDD differentially expressed proteins. Importantly, we also observe an upregulation of CRH signaling in PTSD brain highlighting the importance of glucocorticoid signaling after traumatic stress [89–91]. We did find 65 overlapping proteins between DLPFC of this study and mPFC of the Daskalakis et al. for PTSD which we believe to be highly significant with important etiological relevance. Such integrative analysis of multiple data modalities and studies is necessary to understanding these diseases and provide likely points for therapeutic intervention of upstream regulators.

## Conclusions

In conclusion, we present one of the largest human postmortem proteomics studies for PTSD and MDD. This work fills a critical gap bridging transcriptomic and proteomic genomic signatures in postmortem molecular studies. We identified changes in key PTSD proteins and pathways such as GABAergic interneuron singing which included changes to the GABA transporter SLC32A1. Surprisingly, we found only moderate overlap in the proteomic signatures between PTSD and MDD but identified co-expression patterns common to both disorders. Further, we were able to confirm many miRNA-DEP interactions that were predicted by TargetScan and miRbase including CACNA2D1, CNTN1, THY1, OPCML, CD59, NEGR1, and SLC32A1 as targets of the miRNA *hsa-mir-589*. These results confirm GABAergic impairment and synaptic dysfunction identified in previous transcriptomic and epigenetic studies of PTSD and highlight the key proteins involved, along with their paired regulatory miRNAs. Cross disorder analysis of our proteomics dataset with recent GWAS studies also identified ASD and MDD risk variant enrichment in PTSD co-expression networks, suggesting pathophysiological convergence and common risk for these disorders. In addition, this large proteomics dataset serves as a rich resource for functional genomics and translational architecture in different human cortical brain regions. Together, these efforts will lead to progress in development of novel therapeutics and treatments for PTSD and depression.

## Supplementary Information

Additional file 1: Figures S1-S18.

Additional file 2: Table S1 is the full list of differential expression results for proteomics data.

Additional file 3: Table S2 is pathway enrichment results of DEPs for MDD and PTSD in DLPFC and sgPFC from Ingenuity Pathway Analysis (IPA).

Additional file 4: Table S3 is shared differentially expressed proteins, genes and pathways between this study and previously published studies.

Additional file 5: Table S4 is protein, RNA and miRNA WGCNA results.

Additional file 6: Table S5 is SynGO analysis results for MDD or PTSD associated protein modules.

Additional file 7: Table S6 is differential expression statistics for miRNA analysis.

Additional file 8: Table S7 is the full miRNA GSEA pathway enrichment results.

Additional file 9: Table S8 is miRNA-protein expression correlation and target information.

Additional file 10: Table S9 is miRNA-proteomic module interactions.

## Data Availability

The raw data that support the findings of this study are available from the National PTSD Brain Bank, but restrictions apply to the availability of these data, which were generated under a DUA for the current study and so are not publicly available. The raw data are available through the National PTSD Brain Bank. Interested investigators should submit dataset requests to the corresponding author and https://www.research.va.gov/programs/tissue_banking/ptsd/ and reference this paper for more information. Applications for the data may be submitted at any time and are reviewed monthly. The processed proteomics, RNA-seq, and miRNA data supporting the conclusions of this article are available in the GitHub repository, https://github.com/mjgirgenti/PTSDCorticalProteomics/tree/main/data [92]. The GWAS data used in this study are downloaded from Psychiatric Genomics Consortium (PGC) at https://pgc.unc.edu/for-researchers/download-results/. Members of the Traumatic Stress Brain Research Group (Consortia Authors): Victor E. Alvarez, MD, David Benedek, MD, Alicia Che, PhD, Dianne A. Cruz, MS, David A. Davis, PhD, Matthew J. Girgenti, PhD, Ellen Hoffman, MD, PhD, Paul E. Holtzheimer, MD, Bertrand R. Huber, MD, PhD, Alfred Kaye, MD, PhD, John H. Krystal, MD, Adam T. Labadorf, PhD, Terence M. Keane, PhD, Mark W. Logue, PhD, Ann McKee, MD, Brian Marx, PhD, Mark W. Miller, PhD, Crystal Noller, PhD, Janitza Montalvo-Ortiz, PhD, Meghan Pierce, PhD,William K. Scott, PhD, Paula Schnurr, PhD, Krista DiSano, PhD, Thor Stein, MD, PhD, Robert Ursano, MD, Douglas E. Williamson, PhD, Erika J. Wolf, PhD, Keith A. Young, PhD.

## Abbreviations

ACN: Acetonitrile
ALZ: Alzheimer’s disorder
ASD: Autistic spectrum disorder
BEH: Ethylene Bridged Hybrid
BIP: Bipolar disorder
CNS: Central nervous system
CRH: Corticotropin-releasing hormone
CSEA: Cell type-specific enrichment analysis
DDA: Data dependent acquisition
DEG: Differentially expressed gene
DEP: Differentially expressed protein
DIA: Data-independent acquisition
DLPFC: Dorsolateral prefrontal cortex
DNA: Deoxyribonucleic acid
NOS: Not Otherwise Specified
FC: Fold change
FDR: False discovery rate
FPKM: Fragments per kilobase of transcript per million mapped reads
GABA: Gamma-aminobutyric acid
GEO: Gene expression omnibus
GTEx: Genotype-tissue expression
HDC: Hydrodynamic chromatography
IPA: Ingenuity pathway analysis
LC: Liquid chromatography
MDD: Major depressive disorder
miRNA: MicroRNA
mRNA: Messenger RNA
MS: Mass spectrometry
NMDA: N-methyl-D-aspartate
PCA: Principal component analysis
PFC: Prefrontal cortex
PMI: Postmortem interval
PrimaryDx: Primary diagnosis
PTSD: Post-traumatic stress disorder
RIPA: Radioimmunoprecipitation assay
RIN: RNA integrity number
RNA: Ribonucleic acid
RNA-seq: RNA sequencing
RRHO: Rank-rank hypergeometric overlap
SCZ: Schizophrenia
sgPFC: Subgenual prefrontal cortex
smRNA-seq: Small RNA sequencing
TFA: Trifluoroacetic acid
TRAP: Translating ribosomal affinity purification
TWAS: Transcriptome-wide association study
WGCNA: Weighted gene co-expression network analysis
