# Supplementary material for: A multi-omic approach implicates novel protein dysregulation in post-traumatic stress disorder
Source: Genome Med. 2025 Apr 29;17:43. doi: 10.1186/s13073-025-01473-1 (PMC12042318; doi:10.1186/s13073-025-01473-1)
Supplement: Supplementary file 1 — Additional file 1: Figures S1-S18. [file 13073_2025_1473_MOESM1_ESM.pdf]

Fig. S1

A

Demographics of Proteomic (Mass Spectrometry) donors

	CON	MDD	PTSD	P-value (PTSD vs CON)	P-value (PTSD vs MDD)
N	19	19	19	NA	NA
RIN (miRNA): dlPFC	6.9 ± 1.2	7.4 ± 1.3	7.8 ± 1.0	0.032	0.4
RIN (miRNA): sgPFC	8.0 ± 1.1	8.2 ± 0.8	8.2 ± 1.1	0.54	0.96
Sex (%Male)	51.4	48.6	48.6	1	1
Ethnicity (%White)	71.4	83.8	78.3	0.68	0.77
Age at death	49.8 ± 10.6	47.9 ± 11.6	47.4 ± 10.9	0.32	0.85
Postmortem interval (PMI)	18.4 ± 6.5	16.7 ± 5.6	15.7 ± 5.8	0.06	0.41
Comorbid depression (%)	0	100	45.9	$1.6x10^{-5}$	$6.6x10^{-7}$
Tobacco (%ATOD)	31.4	27	62.2	0.017	0.005
Antidepressants (%ATOD)	0	75.7	67.6	$7.9x10^{-9}$	0.61
Alcohol	0	21.6	35.1	$3.6x10^{-4}$	0.3
Opioid	0	27	35.1	$3.6x10^{-4}$	0.62
Manner of death					
Suicide (%)	0	21.6	5.4	0.09	0.5
Natural (%)	77.1	43.2	56.8	0.11	0.35
Accident (%)	17.1	35.1	32.4	0.22	1

B

Demographics of transcriptomics (RNA-seq) donors

	CON	MDD	PTSD	P-value (PTSD vs CON)	P-value (PTSD vs MDD)
N	22	22	22	NA	NA
RIN (miRNA): dlPFC	6.9 ± 1.2	7.4 ± 1.3	7.7 ± 0.9	0.024	0.33
RIN (miRNA): sgPFC	8.1 ± 1.0	8.2 ± 0.8	8.2 ± 1.1	0.71	0.99
Sex (%Male)	50	50	50	1	1
Ethnicity (%White)	77.3	81.8	81.8	0.51	1
Age at death	47.8 ± 11.7	45.9 ± 12.34	46.0 ± 11.4	0.88	0.89
Postmortem interval (PMI)	18.8 ± 6.8	17.0 ± 5.6	16.0 ± 5.6	0.087	0.49
Comorbid depression (%)	0	100	50	$7.4x10^{-7}$	$7.8x10^{-7}$
Tobacco (%ATOD)	27.2	22.7	63.6	0.034	0.009
Antidepressants (%ATOD)	0	76.2	65.8	$2.1x10^{-9}$	0.43
Alcohol	0	16.7	43.9	$9.3x10^{-6}$	0.014
Opioid	0	28.6	26.8	$1.6x10^{-3}$	1
Manner of death					
Suicide (%)	0	22.7	9.1	0.14	0.16
Natural (%)	69.2	40.5	58.5	0.45	0.15
Accident (%)	25.6	35.7	26.8	1	0.52

C

Demographics of smRNA-seq donors

	**CON**	**MDD**	**PTSD**	**P-value (PTSD vs CON)**	**P-value (PTSD vs MDD)**
N	18	22	17	NA	NA
RIN (miRNA): dlPFC	6.9 ± 1.2	7.4 ± 1.3	7.8 ± 1.0	0.40	0.032
RIN (miRNA): sgPFC	8.0 ± 1.1	8.2 ± 0.8	8.2 ± 1.1	0.96	0.54
Sex (%Male)	62.9	51.2	46.9	0.29	0.89
Ethnicity (%White)	71.4	82.9	84.3	0.33	1
Age at death	45.9 ± 12.1	46.7 ± 12.0	47.3 ± 11.5	0.83	0.63
Postmortem interval (PMI)	17.6 ± 5.3	15.0 ± 4.9	16.8 ± 5.7	0.16	0.038
Comorbid depression (%)	0	100	56.3	9.0x10-7	1.0x10-5
Tobacco (%ATOD)	22.9	51.2	46.9	0.011	0.0056
Antidepressants (%ATOD)	0	68.8	31.3	1.0x10-8	0.53
Alcohol	0	37.5	68.8	2.3x10-4	0.088
Opioid	0	28.1	29.3	2.6x10-3	1
Manner of death					
Suicide (%)	0	24.3	12.5	0.10	0.33
Natural (%)	65.7	39.0	59.4	0.78	0.14
Accident (%)	28.6	36.6	21.9	0.73	0.27

D **E**

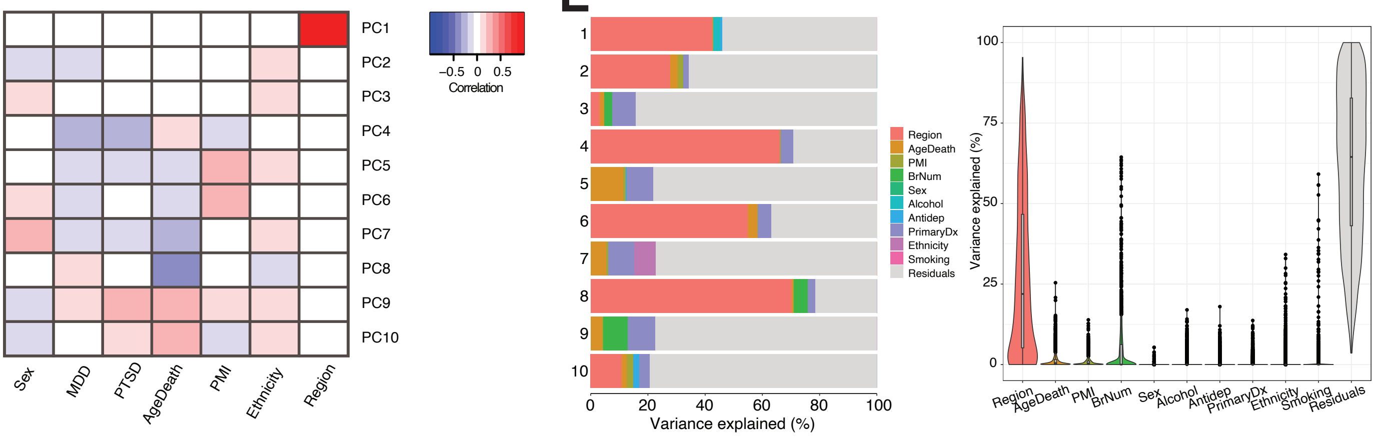

Fig. S1 | Demographics information of donors for the three omics datasets and preprocessing. (**A**) Demographics table for proteomics donors. (**B**) Demographics for transcriptomics (RNA-seq) donors. (**C**) Demographic table for smRNA-seq donors. (**D**) Effects of selected traits in top PC space. (**E**) Variance partition analysis of variance of protein abundance.

Fig. S2

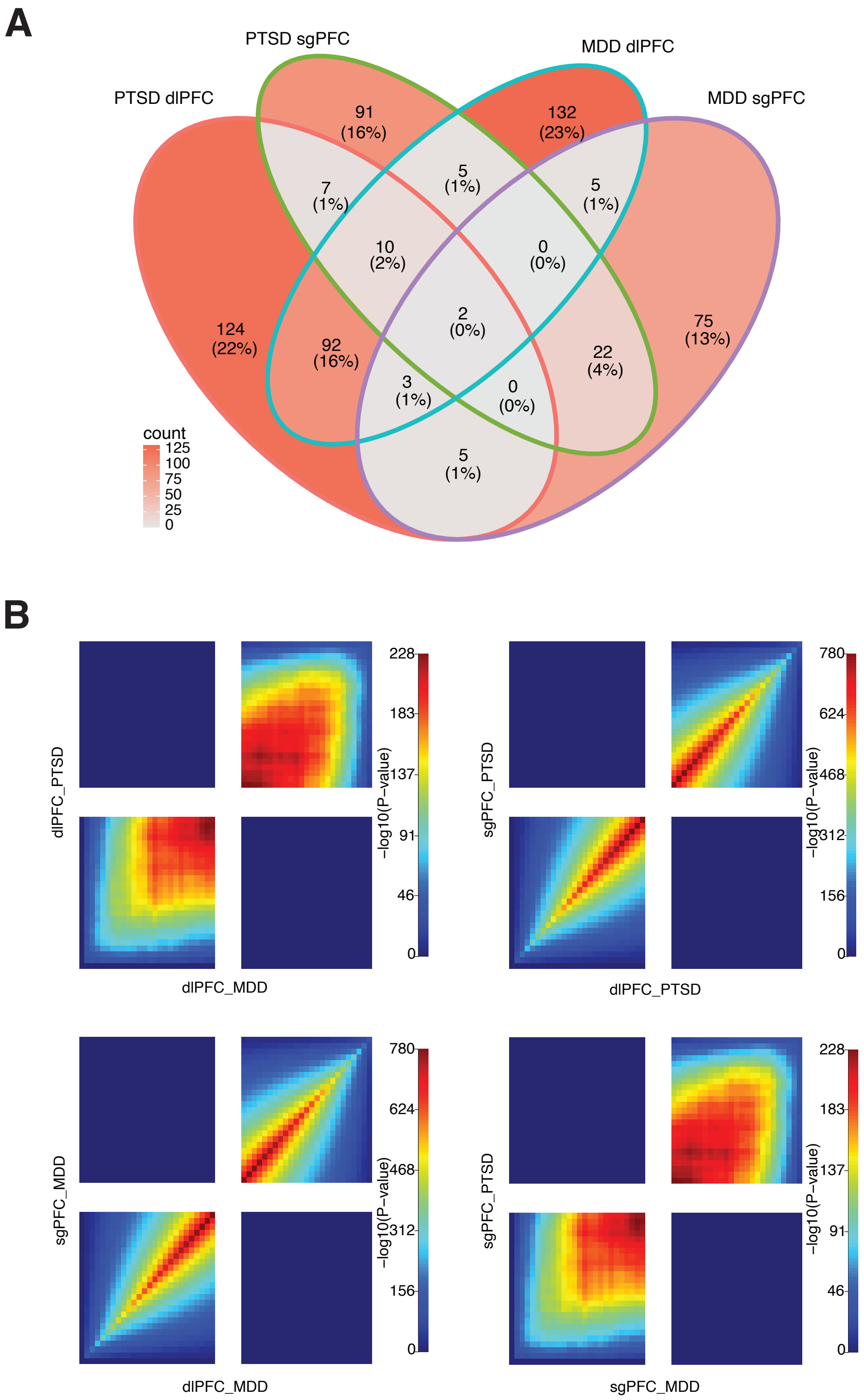

Fig. S2 I Protein differential expression analysis. (**A**) Venn diagram showing overlap of DEPs (nominal $P <$ 0.05) between regions and diagnostic cohorts. (**B**) RRHO plots of protein differential expression concordance.

Fig. S3

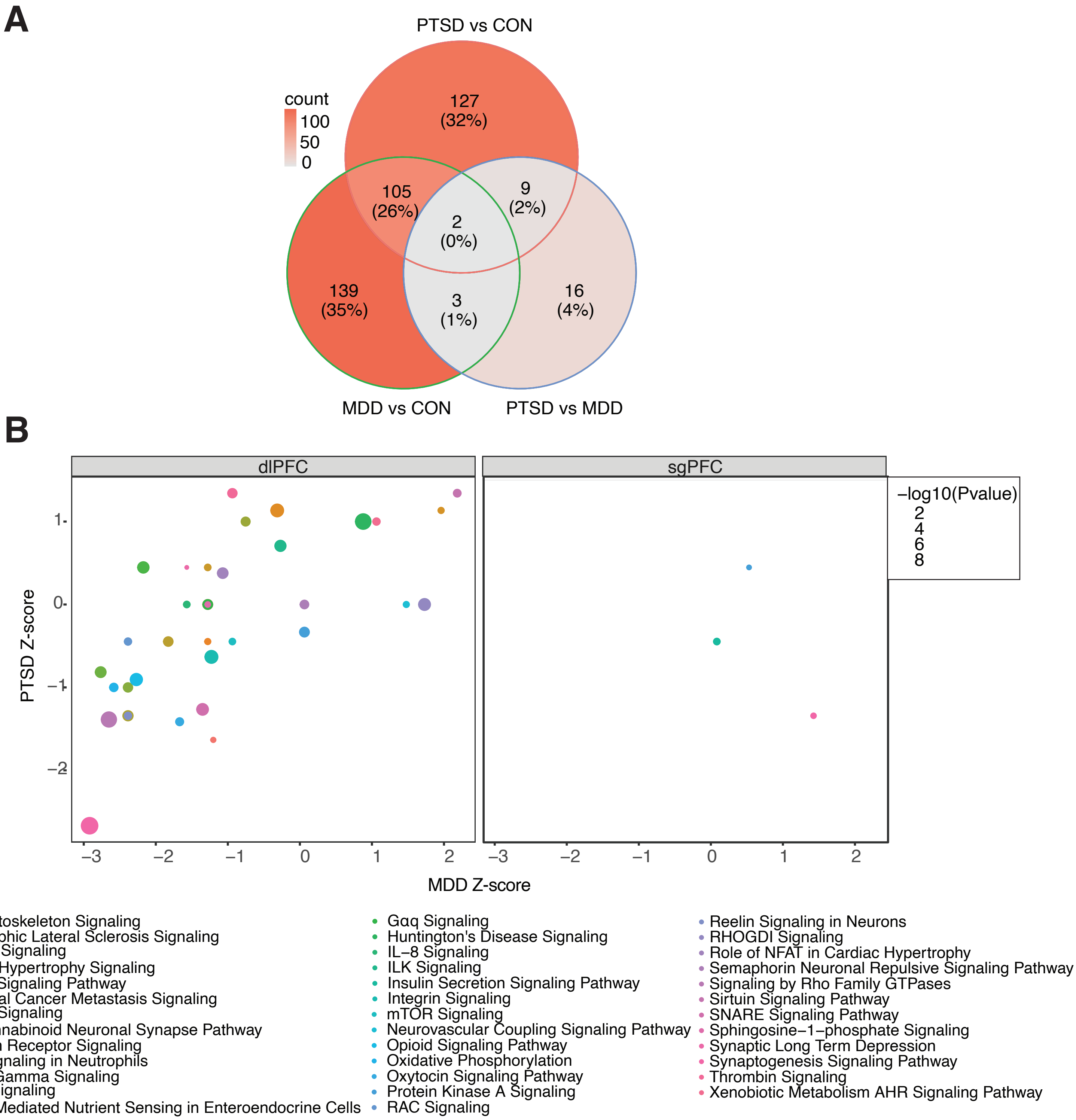

Fig. S3 | Common and divergent proteomic signatures between MDD and PTSD. (**A**) Venn diagrams show the overlap of DEPs between CON, MDD and PTSD groups in DLPFC (P<0.05). (**B**) Comparison of the changing gene set enrichment (Z-scores) and directions of the biological pathways between MDD and PTSD in DLPFC and sgPFC. In DLPFC, MDD and PTSD share similar changes of pathways with an R^2 = 0.57 while in sgPFC only three pathways were shared. Colors indicate gene set and circle size indicates -log10 (P value).

Fig. S4

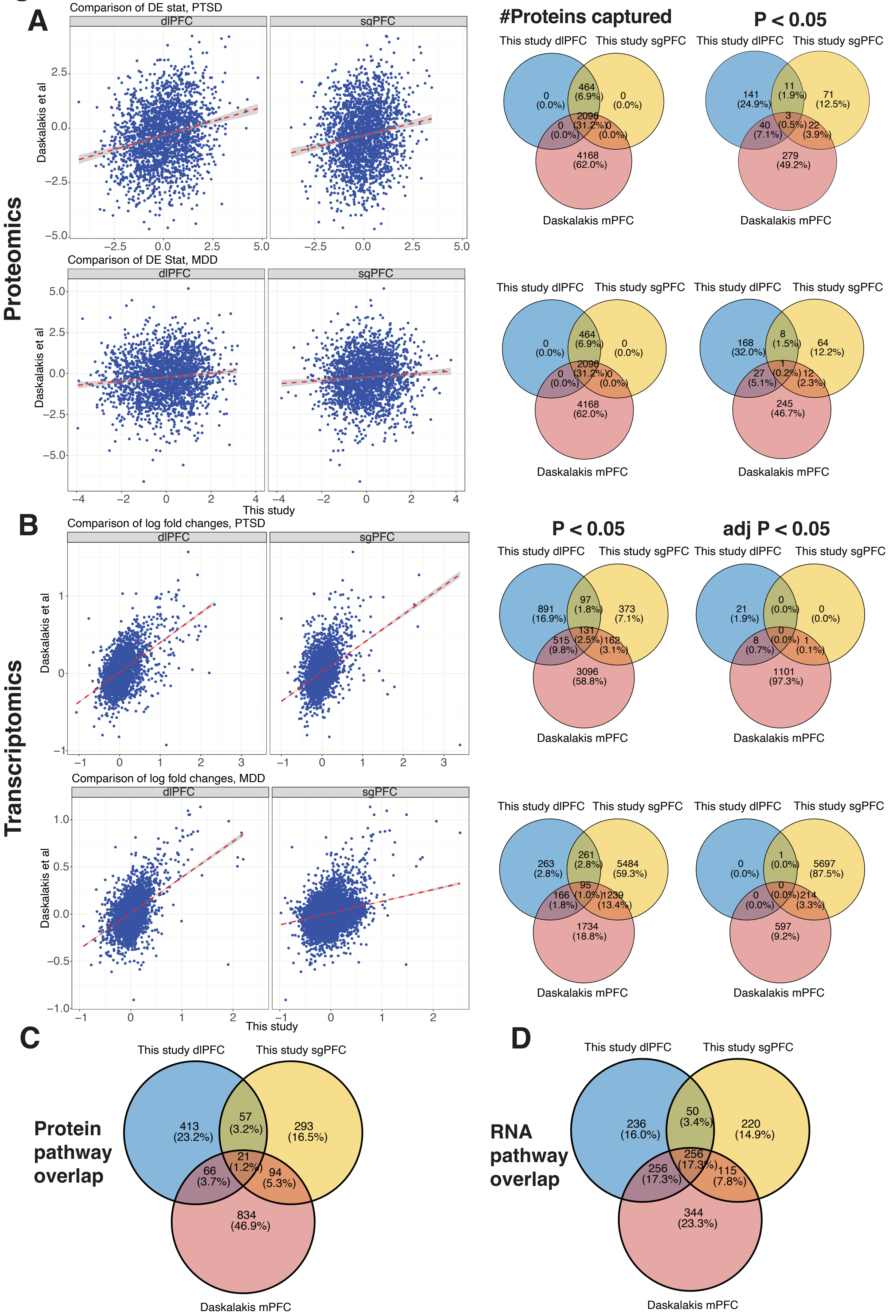

A
Proteomics
Comparison of DE stat, PTSD
dlPFC
sgPFC
Daskalakis et al
#Proteins captured
This study dlPFC
This study sgPFC
Daskalakis mPFC
0 (0.0%)
464 (6.9%)
0 (0.0%)
2096 (31.2%)
0 (0.0%)
0 (0.0%)
4168 (62.0%)
P < 0.05
141 (24.9%)
11 (1.9%)
71 (12.5%)
3 (0.5%)
40 (7.1%)
22 (3.9%)
279 (49.2%)
Comparison of DE Stat, MDD
168 (32.0%)
8 (1.5%)
64 (12.2%)
1 (0.2%)
27 (5.1%)
12 (2.3%)
245 (46.7%)
This study
B
Transcriptomics
Comparison of log fold changes, PTSD
P < 0.05
adj P < 0.05
891 (16.9%)
97 (1.8%)
373 (7.1%)
131 (2.5%)
515 (9.8%)
162 (3.1%)
3096 (58.8%)
21 (1.9%)
0 (0.0%)
0 (0.0%)
0 (0.0%)
8 (0.7%)
1 (0.1%)
1101 (97.3%)
Comparison of log fold changes, MDD
263 (2.8%)
261 (2.8%)
5484 (59.3%)
95 (1.0%)
166 (1.8%)
1239 (13.4%)
1734 (18.8%)
0 (0.0%)
1 (0.0%)
5697 (87.5%)
0 (0.0%)
0 (0.0%)
214 (3.3%)
597 (9.2%)
C
Protein pathway overlap
413 (23.2%)
57 (3.2%)
293 (16.5%)
21 (1.2%)
66 (3.7%)
94 (5.3%)
834 (46.9%)
D
RNA pathway overlap
236 (16.0%)
50 (3.4%)
220 (14.9%)
256 (17.3%)
256 (17.3%)
115 (7.8%)
344 (23.3%)

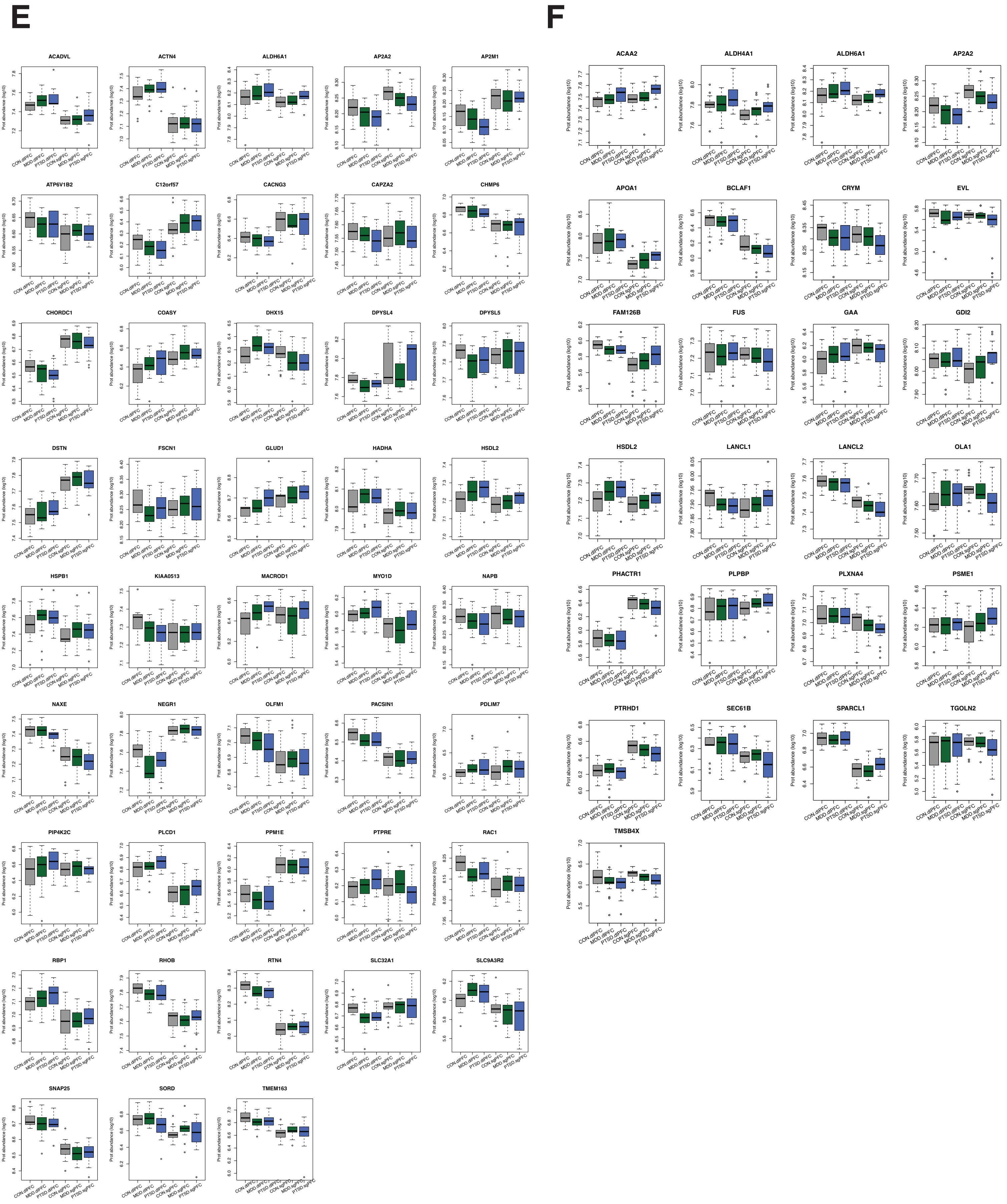

Fig. S4 | Comparison between multiomics results from this study and Daskalakis *et al.* in PTSD and MDD. (**A**) Left, scatterplots showing comparisons of protein differential statistics in response to PTSD (top) or MDD (bottom) in brain region DLPFC or sgPFC. Middle, Venn Diagram showing overlap of proteins captured in each brain region of the two studies. Right, Venn Diagram showing overlap of differentially expressed proteins (*P*-value < 0.05). (**B**) Left, scatterplots showing comparisons of gene differential expression log fold changes in response to PTSD (top) or MDD (bottom) in brain region DLPFC or sgPFC. Middle, Venn Diagram showing overlap of differentially expressed genes (*P*-value < 0.05). Right, Venn Diagram showing overlap of differentially expressed genes (adjusted *P*-value < 0.05). (**C,D**) Venn Diagram showing overlap of significantly enriched pathways in DEPs (**C**) and DEGs (**D**). (**E,F**) Boxplot of abundance levels of 65 overlapped DEPs between mPFC of Daskalakis *et al.* and DLPFC (**E**) or sgPFC (**F**) of this study in PTSD.

Fig. S5

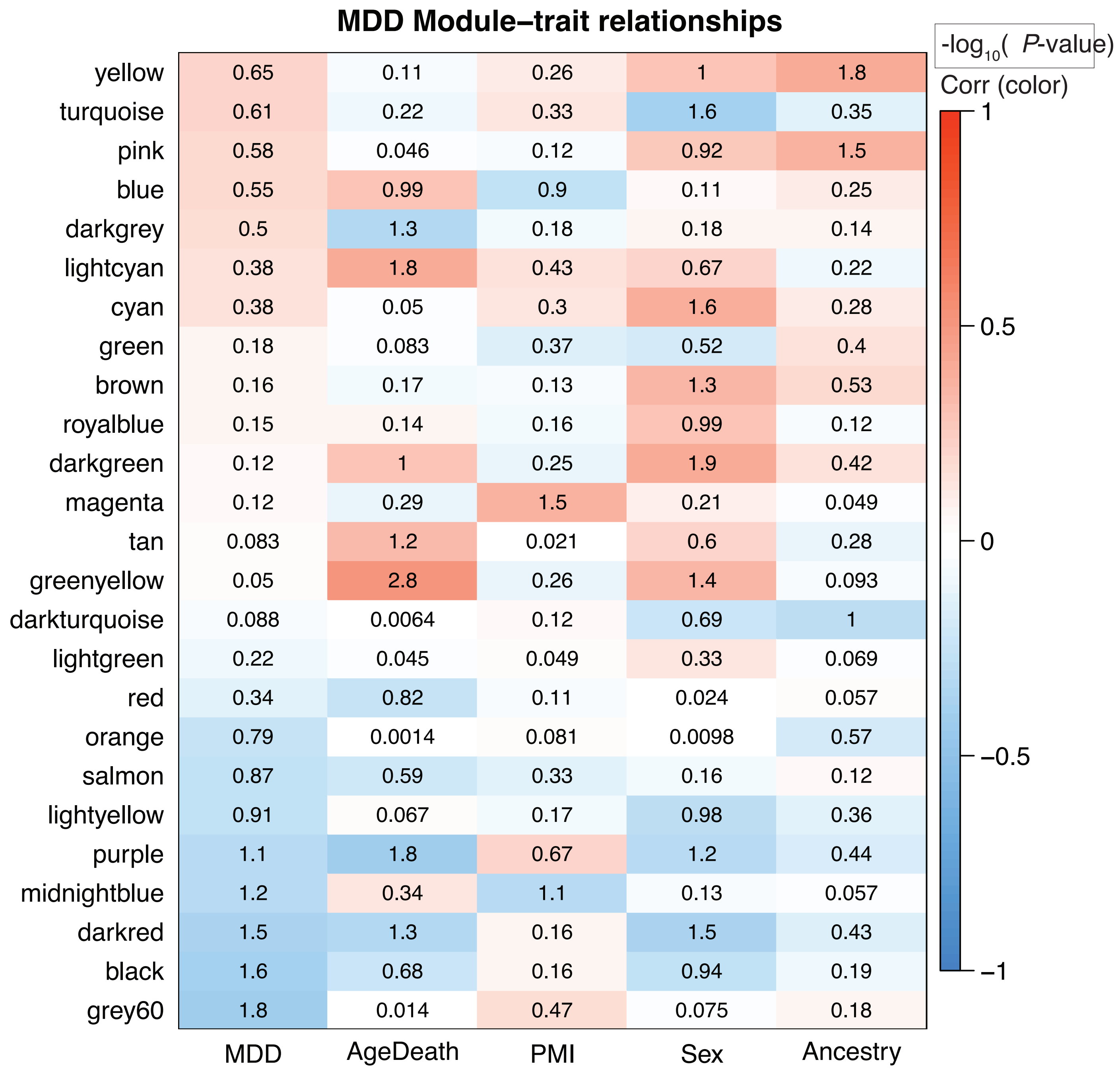

Fig. S5 | Protein module-trait association for MDD. Color in each cell reflects correlation between module eigenprotein and PrimaryDx, while the number represents -$\log_{10}$(*P*-value) of that correlation. Module names are abbreviated as color codes only.

Fig. S6

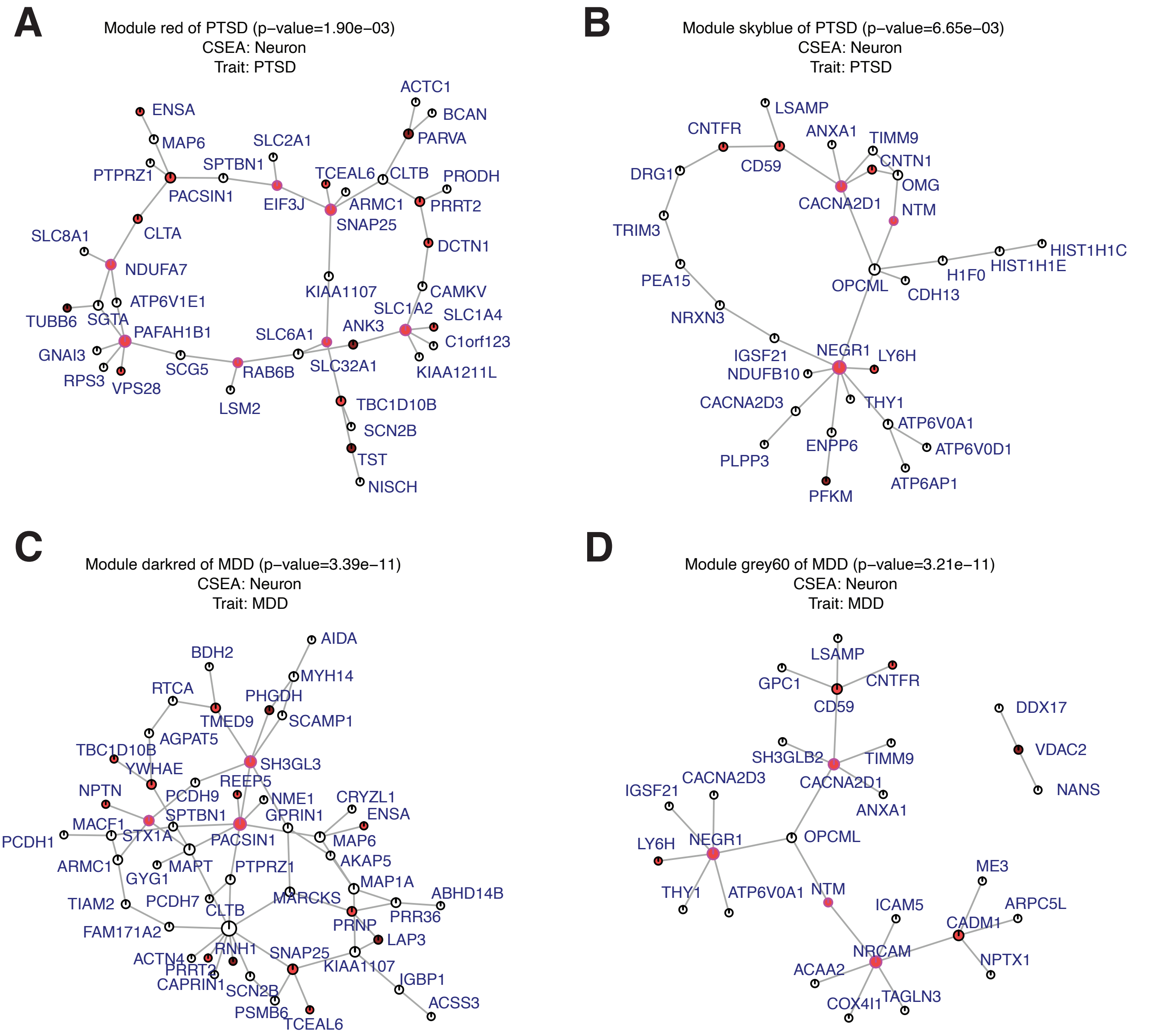

Fig. S6 I Network and key driver analysis of protein modules. KDA plots for module PTSD-PM-*red* (**A**), module PTSD-PM-*skyblue* (**B**), module MDD-PM-*darkred* (**C**), and module MDD-PM-*grey60* (**D**). Key drivers are colored in pink and other nodes are in black. Connections indicate significant associations between two proteins. Node sizes measure their total connectivity. Module names are abbreviated as color codes only.

Fig. S7

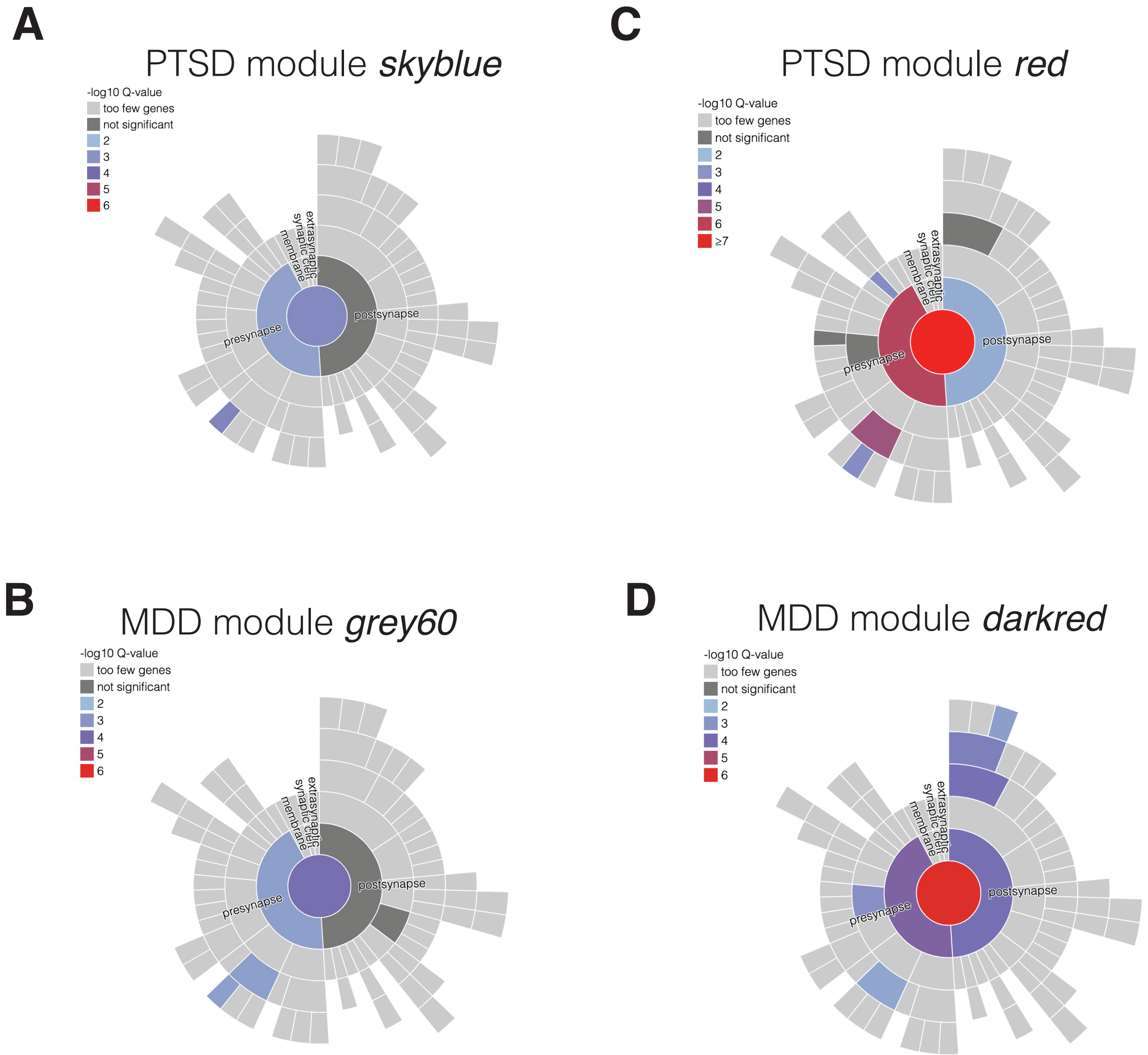

Fig. S7 I Synaptic function enrichment analysis of protein modules. SynGO plots for (**A**) module PTSD-PM-*skyblue*, (**B**) module PTSD-PM-*red*, (**C**) module MDD-PM-*grey60*, (**D**) module MDD-PM-*darkred*. Color legends indicate level of enrichment. Mode SynGO results and statistics are included in Additional file 3: Table S2. Module names are abbreviated as color codes only.

Fig. S8

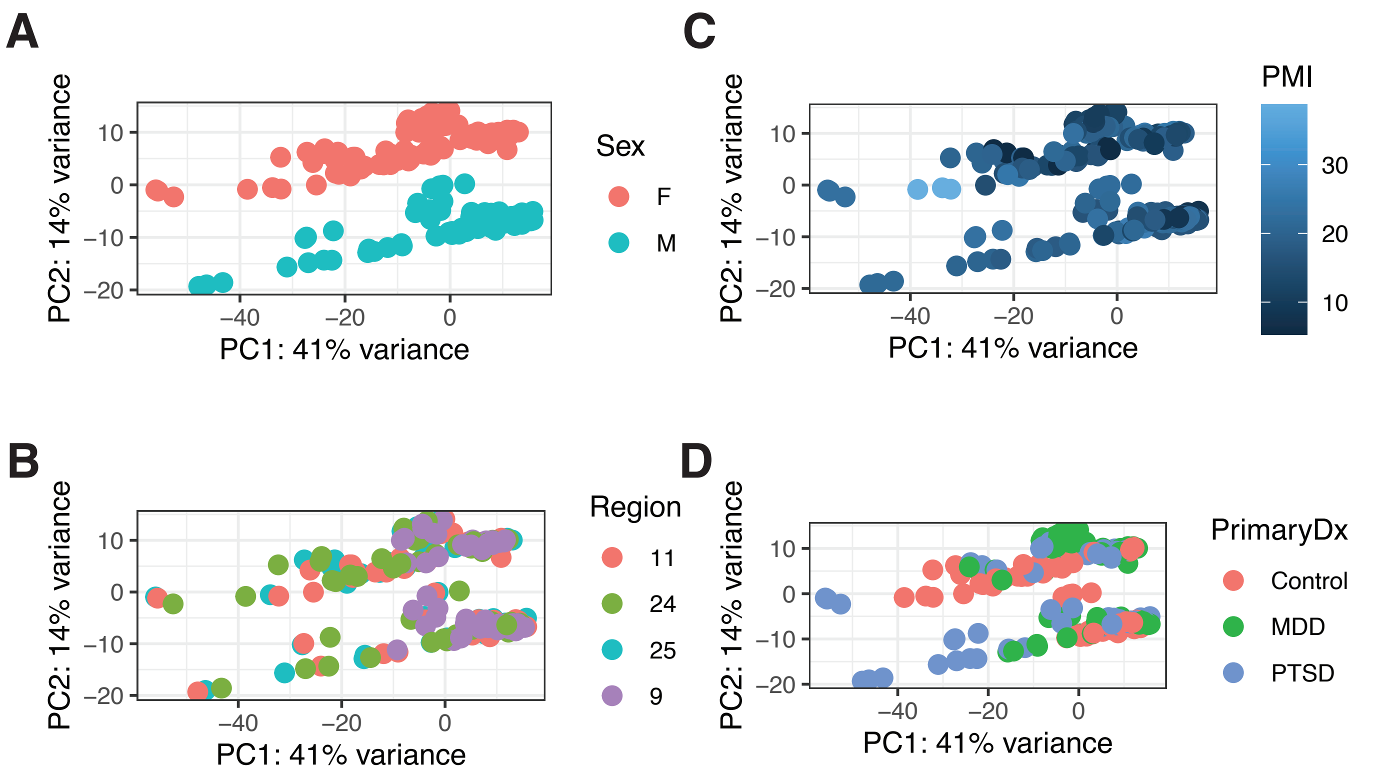

Fig. S8 | PCA plots showing top variance-drivers in transcriptomics data. PCA revealed regional and sex differences that correlated with top principal components and accounted for most variance among all demographic factors (**A**, sex; **B**, PMI; **C**, brain region; **D**, PrimaryDx).

Fig. S9

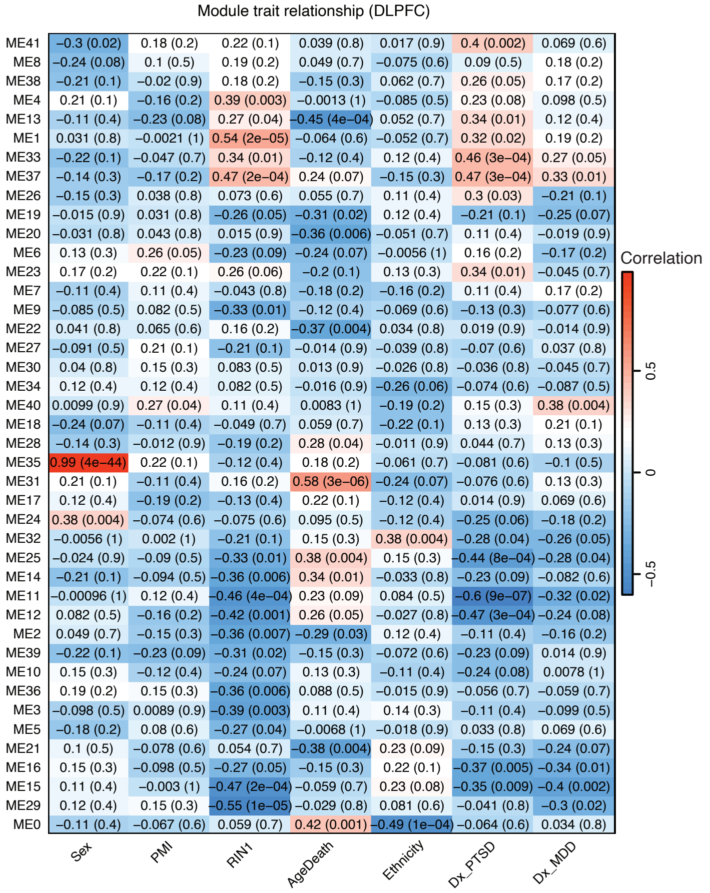

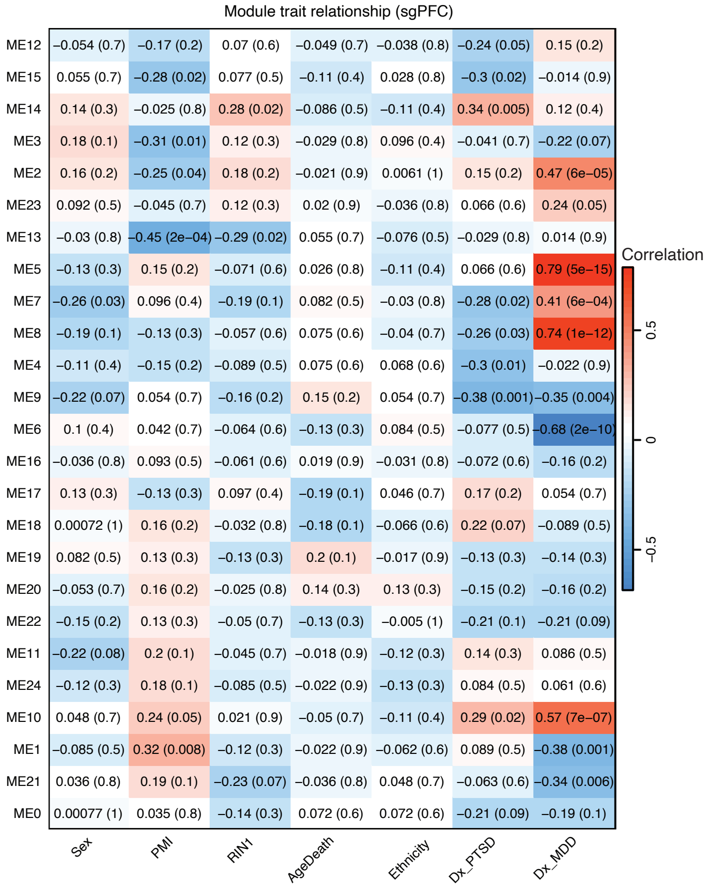

Fig. S9 | Gene co-expression modules and their correlations with demographic traits in DLPFC and sgPFC for PTSD and MDD. Module names are abbreviated as indices only.

Fig. S10

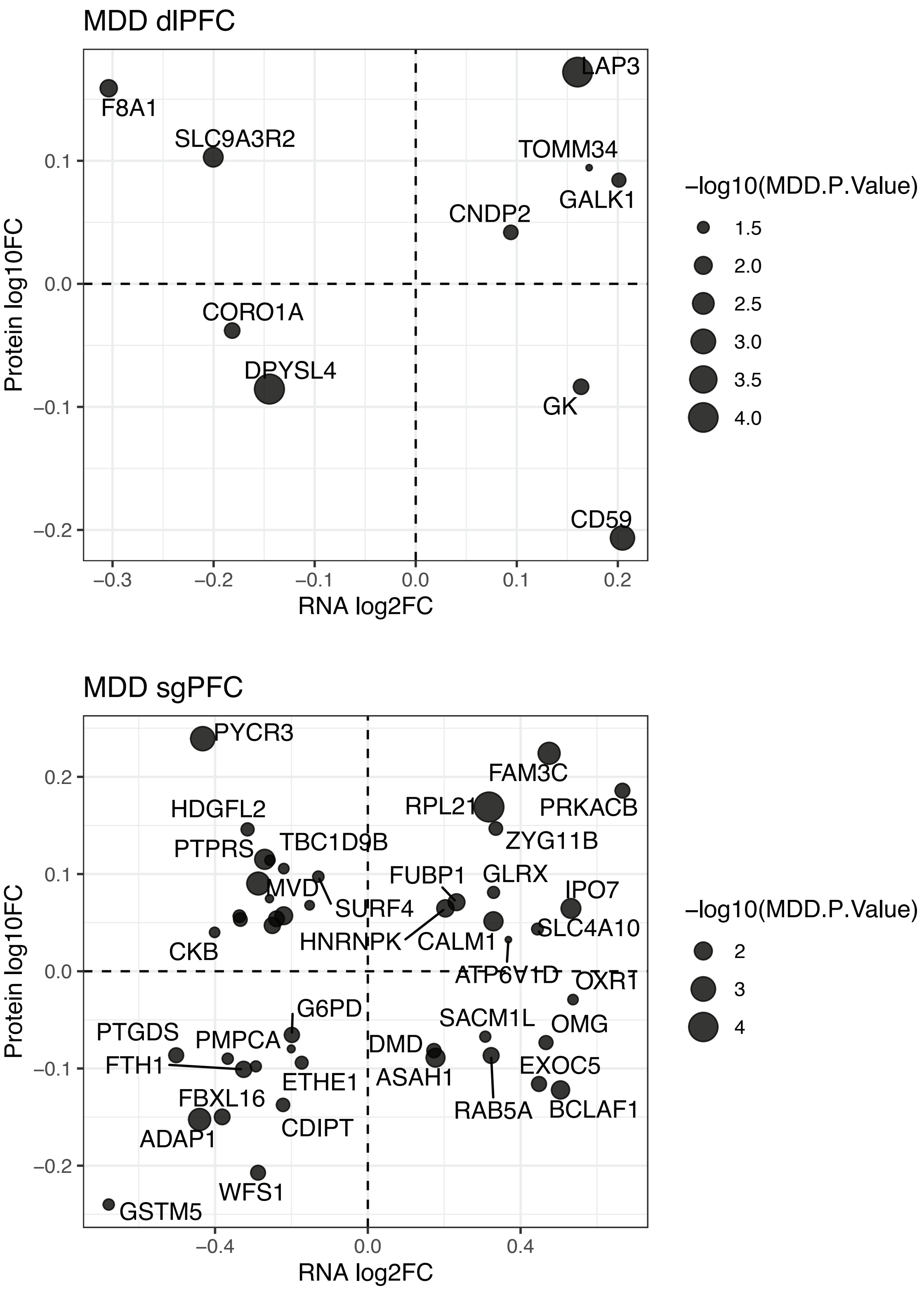

Fig. S10 | Convergence between transcript and protein differential expression in MDD. Top: DLPFC; bottom: sgPFC (bottom).

Fig. S11

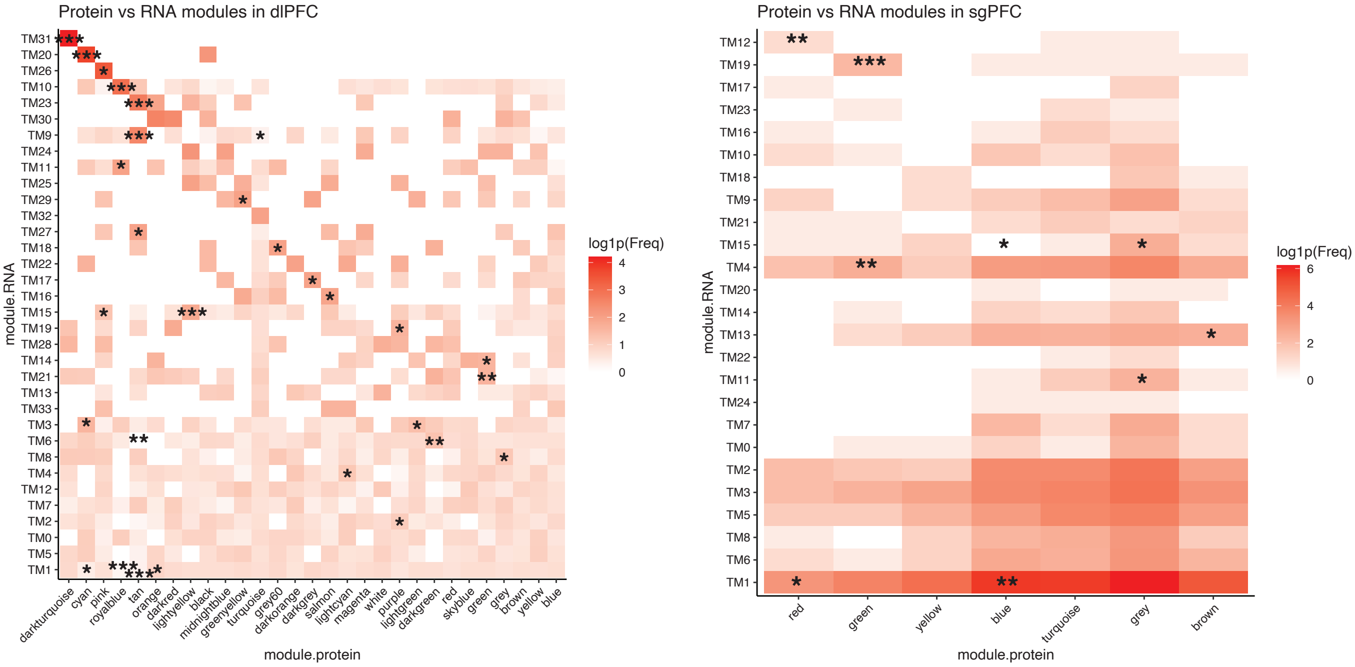

Fig. S11 | Preservation between RNA and protein modules. Asterisks indicate the significance levels of enrichment: ***, *P*-value < 0.001; **, *P*-value < 0.01; *, *P*-value < 0.05.

Fig. S12

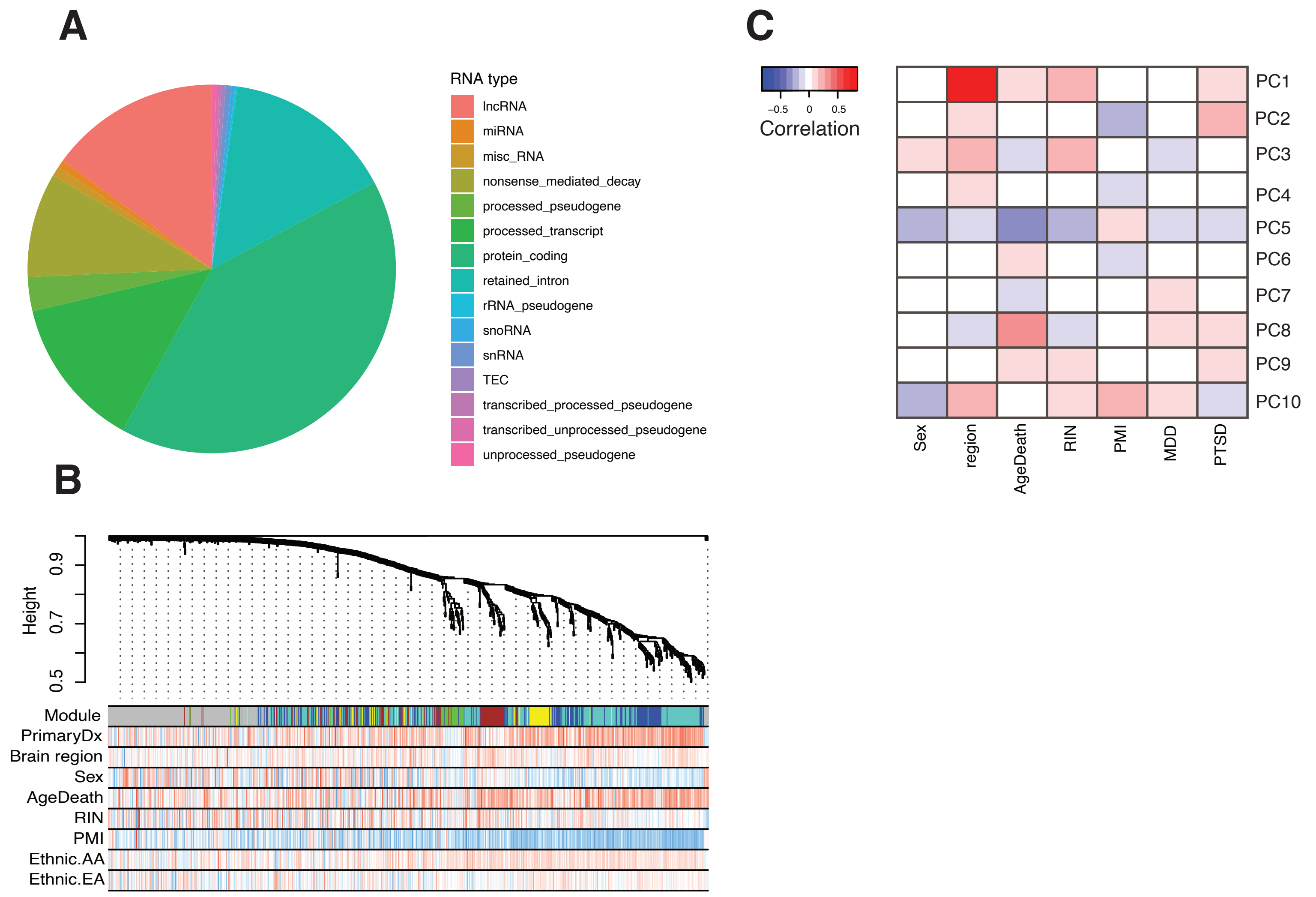

Fig. S12 | Quality control and cluster analysis of miRNA samples. (**A**) smRNA-seq decomposition of RNA types. (**B**) miRNA hierarchical clustering dendrogram. (**C**) PCA of miRNAs samples shows brain region is the most significant trait ($P < 2.2 \times 10^{-16}$ and cor = 0.82) compared to other demographic features (sex, age, RIN, PMI) and diagnosis.

Fig. S13

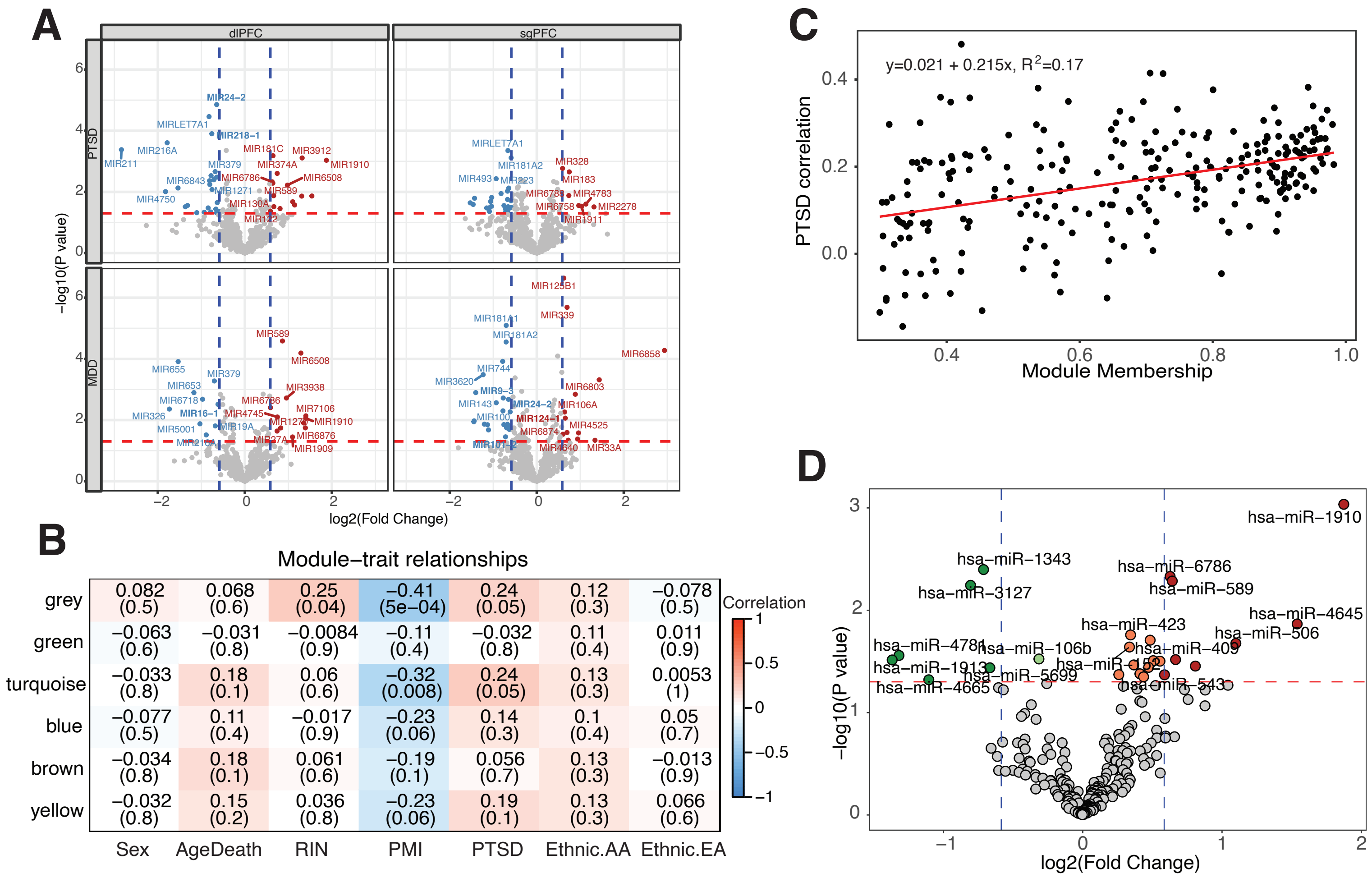

Fig. S13 | Differential expression and network analysis of miRNA samples. (**A**) Region-specific miRNA volcano plots. Vertical blue dashed lines indicate log fold change threshold of $\log_2 FC = \pm\log_2(1.5)$. Horizontal red dashed lines indicate significant threshold $P = 0.05$. (**B**) miRNA WGCNA modules. Colors and numbers (out of parentheses) of cells in the heatmap indicate the correlation between the expression module eigengene and trait, while numbers inside parentheses are P-values of the correlation. (**C**) module MiM-turquoise associated with PTSD with an $R^2 = 0.17$ between the module membership and gene-PTSD diagnosis correlation. (**D**) differential expression of module MiM-turquoise. Vertical dashed lines indicate log fold change threshold of $\log_2 FC = \pm\log_2(1.5)$. Horizontal red dashed lines indicate significance threshold $P = 0.05$. DE miRNAs are marked on the plot as orange (fold change > 1 and $P < 0.05$) and red (fold change > 1.5 and $P < 0.05$), indicating up-regulated miRNAs, and green (fold change < 1 and $P < 0.05$) and darkgreen (fold change < 1/1.5 and $P < 0.05$), indicating down regulated ones. Module names are abbreviated as color codes only.

Fig. S14

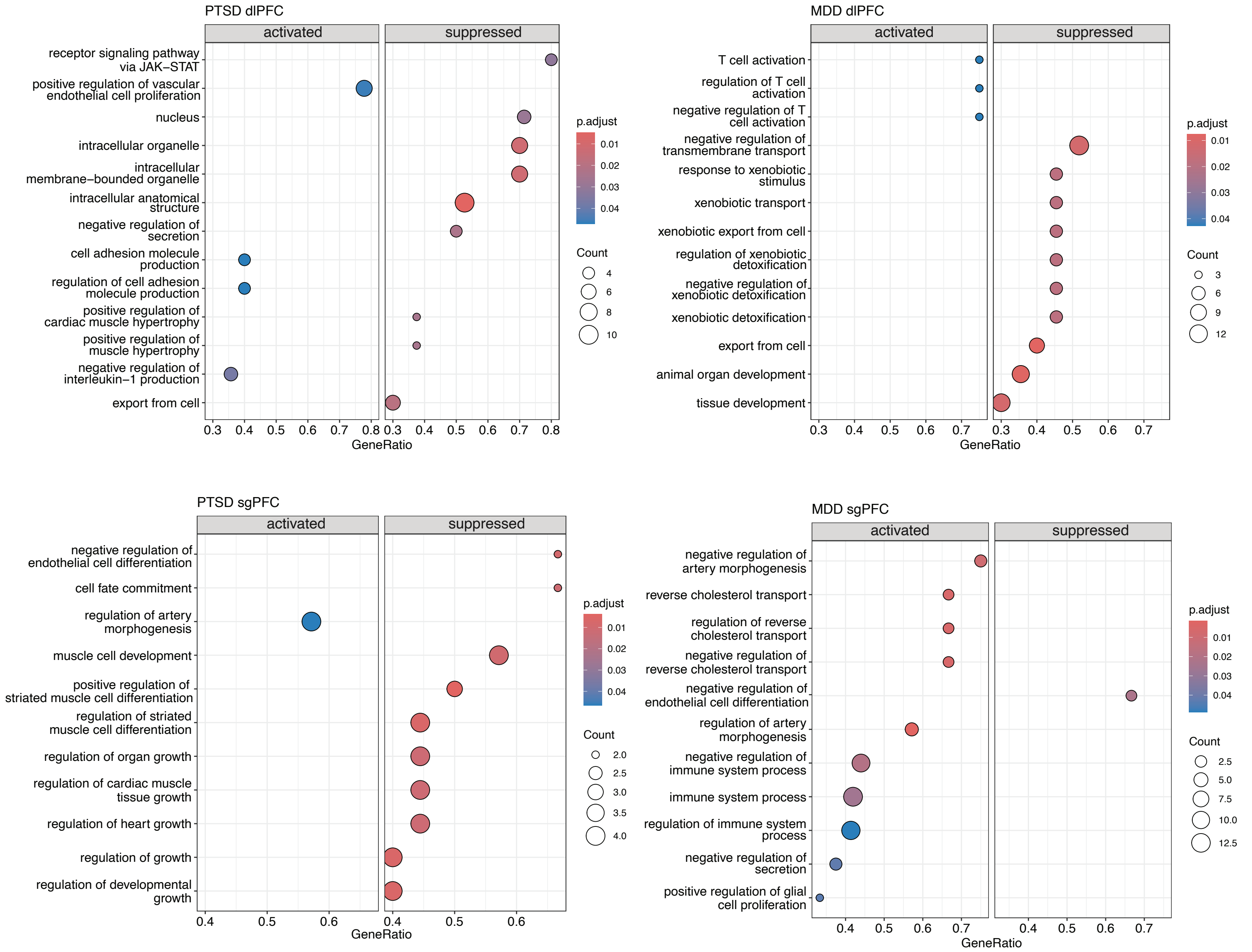

Fig. S14 | GSEA analysis of differentially enriched miRNAs. Scatterplots showing results of pathway enrichment analysis with ClusterProfiler by diagnosis, brain region and regulation direction.

Fig. S15

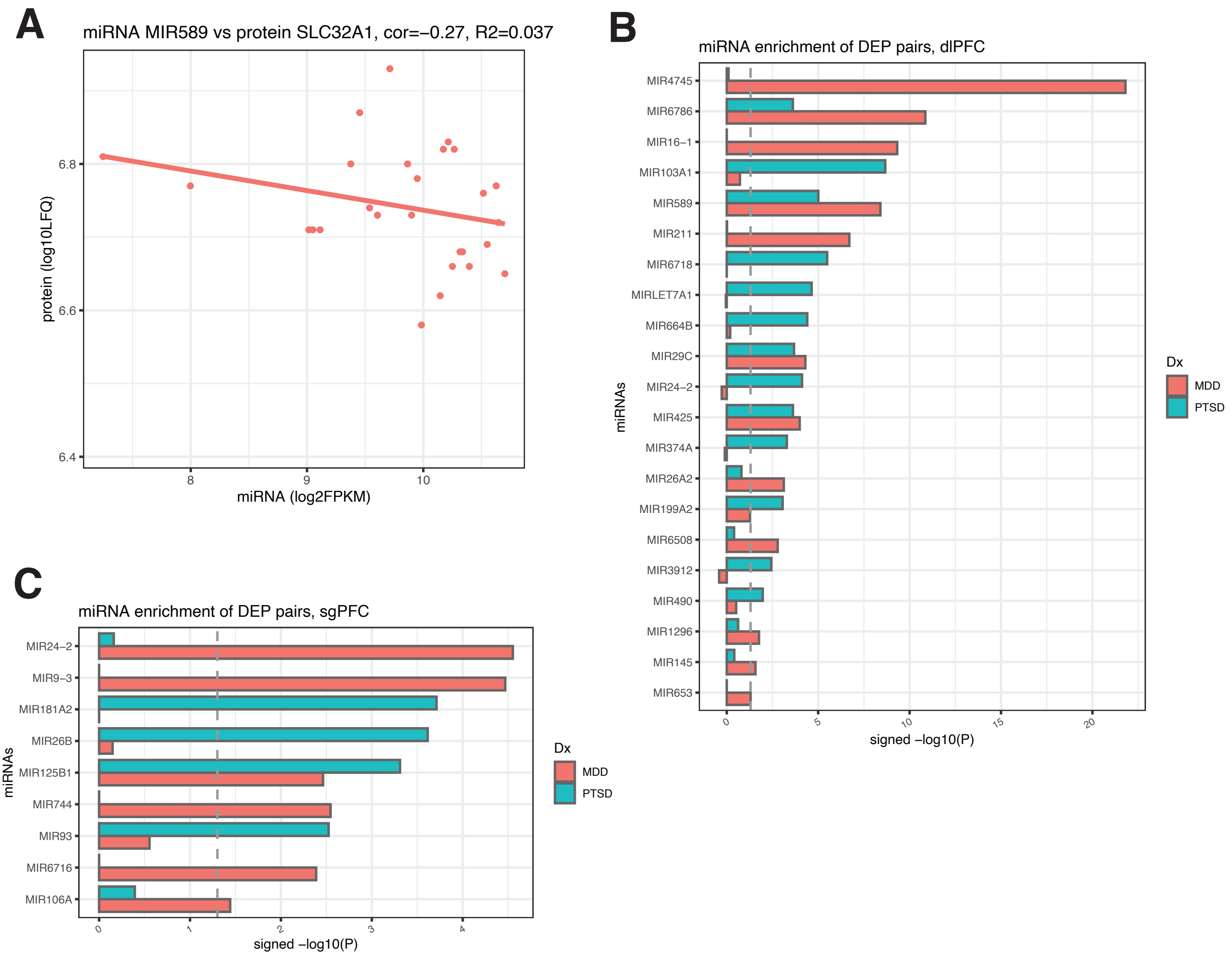

Fig. S15 | miRNA-protein enrichment analysis. (**A**) Sample correlation between *MIR589* and protein LY6H (cor = -0.33 and R^2 = 0.11 and protein SLC32A1 (cor = -0.27 and R^2 = 0.037). (**B,C**) List of miRNA-DEP enrichment scores for MDD (red) and PTSD (blue) in DLPFC (**B**) and sgPFC (**C**). Vertical dashed line indicates significant threshold of $P = 0.05$. miRNA names are abbreviated as “MIR”, e.g. *hsa-mir-4745* as *MIR4745*.

Fig. S16

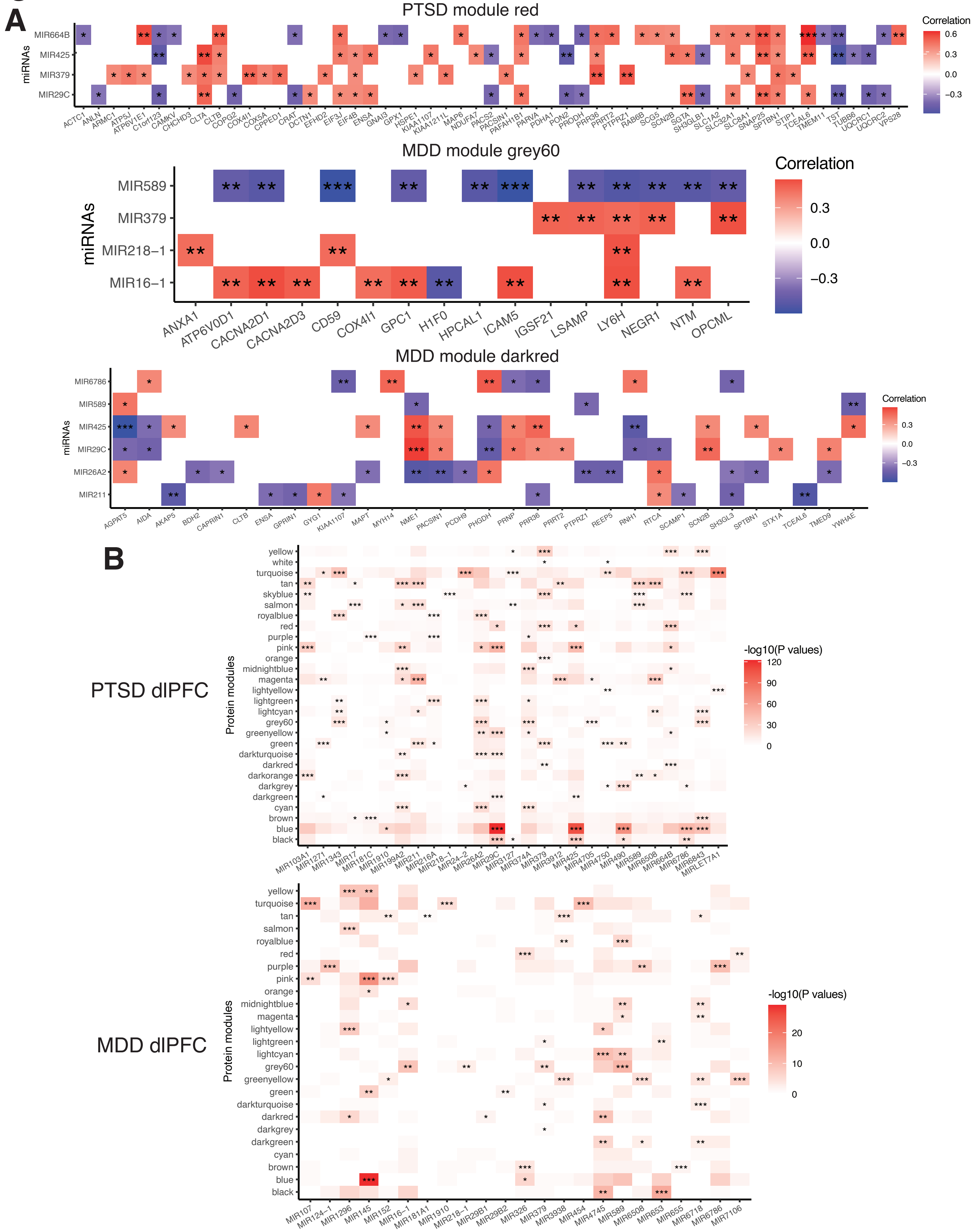

Fig. S16 | miRNA-protein module enrichment analysis. (**A**) miRNA-module correlation in module PTSD-PM-*red* (top), modules MDD-PM-*grey60* (middle) and MDD-PM-*darkred* (bottom). Colors in heatmap indicate the correlation levels between miRNAs and proteins. Plotted are miRNA-protein pairs with *P*-value < 0.05. (**B**) miRNA-module enrichment results for PTSD (top) and MDD (bottom) in DLPFC. Colors of the heatmap indicate the significance levels that are the -$\log_{10}$(*P*-values) of Fisher's exact test enrichment scores. Module names are abbreviated as color codes only. miRNA names are abbreviated as "MIR", e.g. *hsa-mir-29c* as *MIR29C*.

Fig. S17

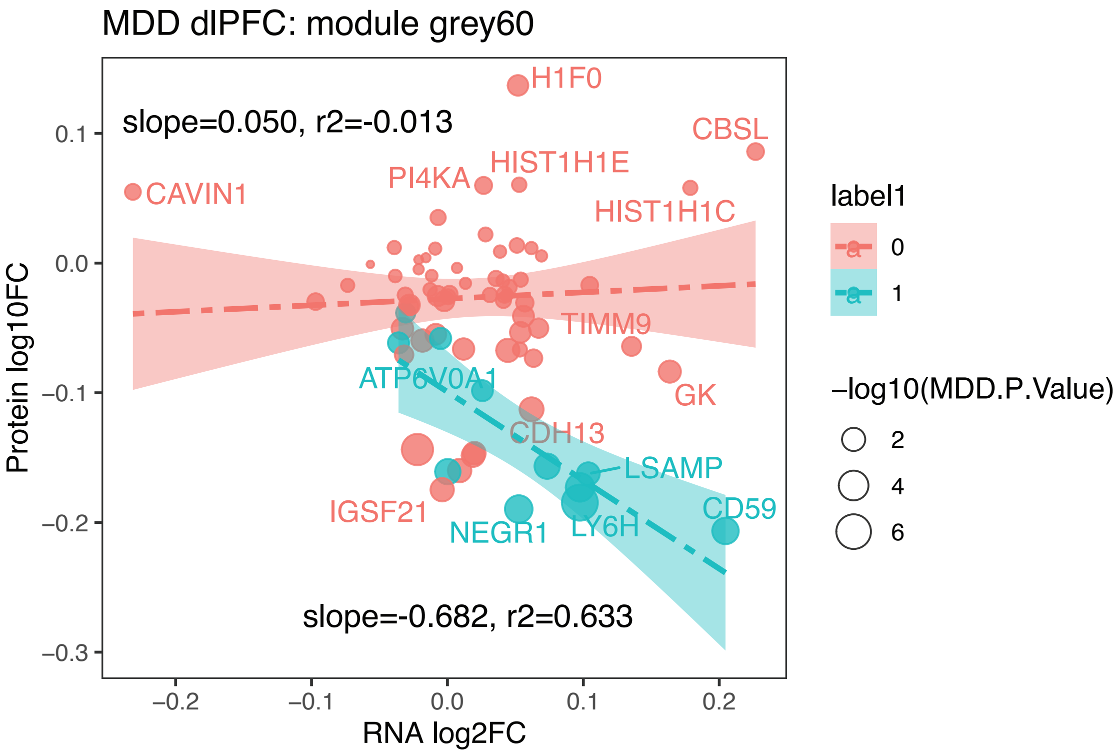

Fig. S17 | Comparison of transcriptional and protein differential expression in MDD module MDD-PM-*grey60*. Module name is abbreviated as color code only.

Fig. S18

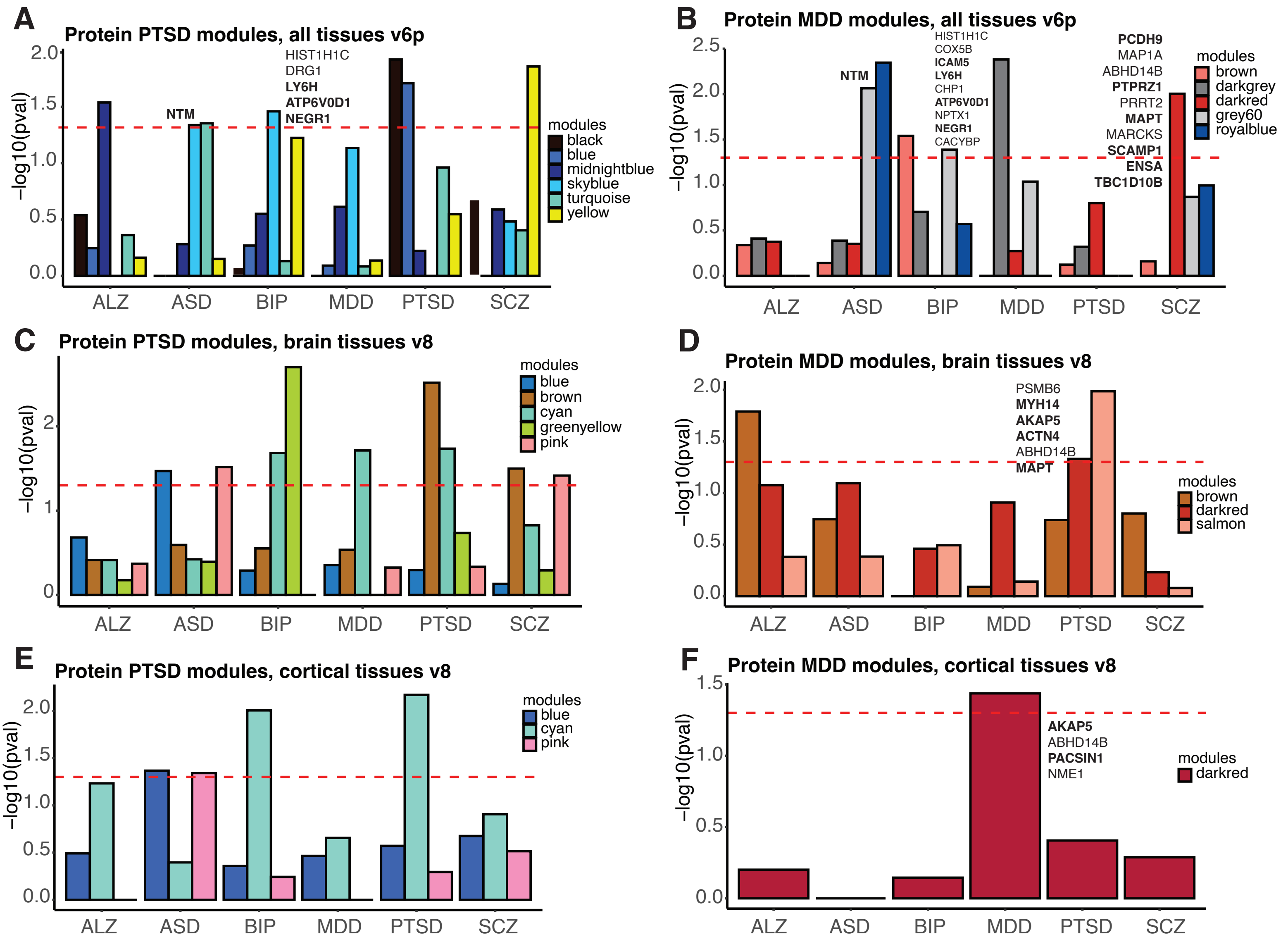

Fig. S18 | Enrichment of GWAS risks of multiple neuropsychiatric traits in protein modules in PTSD and MDD. Enrichment of GWAS risk genes from TWAS analysis in the proteomic modules of PTSD and MDD, including Alzheimer's disease (ALZ), autism spectrum disorder (ASD), bipolar disorder (BIP), major depression disorder (MDD), post-traumatic stress disorder (PTSD) and schizophrenia (SCZ). (**A**) Module enrichment results from all-tissue joint analysis for PTSD modules with GTEx v6p. (**B**) Module enrichment results from all-tissue joint analysis for MDD modules with GTEx v6p. (**C**) Module enrichment results from all-brain tissue joint analysis for PTSD modules with GTEx v8. (**D**) Module enrichment results from all-brain tissue joint analysis for MDD modules with GTEx v8. (**E**) Module enrichment results from all-cortical tissue joint analysis for PTSD modules with GTEx v8. (**F**) Module enrichment results from all-cortical tissue joint analysis for MDD modules with GTEx v8. Overlap between genetic hits and those from modules PTSD-PM-*skyblue*, MDD-PM-*grey60*, MDD-PM-*darkred* were labeled. Bold are DEPs ($P < 0.05$).
